# A biophysical model of dynamic balancing of excitation and inhibition in fast oscillatory large-scale networks

**DOI:** 10.1371/journal.pcbi.1006007

**Published:** 2018-02-23

**Authors:** Romesh G. Abeysuriya, Jonathan Hadida, Stamatios N. Sotiropoulos, Saad Jbabdi, Robert Becker, Benjamin A. E. Hunt, Matthew J. Brookes, Mark W. Woolrich

**Affiliations:** 1 Oxford Centre for Human Brain Activity, Wellcome Centre for Integrative Neuroimaging, Department of Psychiatry, University of Oxford, United Kingdom; 2 Oxford Centre for Functional Magnetic Resonance Imaging of the Brain, Wellcome Centre for Integrative Neuroimaging, University of Oxford, United Kingdom; 3 Sir Peter Mansfield Imaging Centre, School of Physics and Astronomy, University of Nottingham, United Kingdom; 4 National Institute for Health Research (NIHR) Nottingham Biomedical Research Centre, Queens Medical Centre, Nottingham; 5 Department of Diagnostic Imaging, Neurosciences & Mental Health, Research Institute, The Hospital for Sick Children, Toronto, Ontario, Canada; Ghent University, BELGIUM

## Abstract

Over long timescales, neuronal dynamics can be robust to quite large perturbations, such as changes in white matter connectivity and grey matter structure through processes including learning, aging, development and certain disease processes. One possible explanation is that robust dynamics are facilitated by homeostatic mechanisms that can dynamically rebalance brain networks. In this study, we simulate a cortical brain network using the Wilson-Cowan neural mass model with conduction delays and noise, and use inhibitory synaptic plasticity (ISP) to dynamically achieve a spatially local balance between excitation and inhibition. Using MEG data from 55 subjects we find that ISP enables us to simultaneously achieve high correlation with multiple measures of functional connectivity, including amplitude envelope correlation and phase locking. Further, we find that ISP successfully achieves local E/I balance, and can consistently predict the functional connectivity computed from real MEG data, for a much wider range of model parameters than is possible with a model without ISP.

## Author summary

Recently there has been much interest in investigating the role of synaptic plasticity in supporting healthy brain activity. In particular, the balance between excitation and inhibition in the brain is believed to play a critical role in brain dynamics, and it is likely that this balance is regulated by homeostatic mechanisms. Biophysical models of the brain have previously been used to predict functional connectivity, but are typically extremely sensitive to changes in parameter values and require extremely fine tuning to achieve realistic dynamics. In this study, we investigated whether including a homeostatic plasticity mechanism would improve the robustness of simulated neural dynamics. We focused on functional connectivity in MEG data, which can resolve fast oscillations in neural activity, unlike fMRI. We found that including a simple plasticity rule to balance excitation and inhibition resulted in more realistic model predictions, and reduced sensitivity to changes in model parameters.

## Introduction

Healthy resting brain dynamics exhibit several characteristic spatiotemporal features, including structured functional connectivity between brain regions detectable by a variety of different measures. The mechanisms that give rise to this functional connectivity are still under investigation, and large-scale biophysical models offer a parsimonious way to mechanistically explore how brain structure and neuronal properties give rise to functional connectivity. There has been considerable interest in using coupled networks of oscillators to relate large-scale functional connectivity to network properties including connectivity strength, time delays, and graph structure. These oscillator models span very simple oscillators such as the Kuramoto model [1–7], through to more sophisticated oscillators based on Hopf bifurcations [8,9], and biophysical neural mass models such as the Wilson-Cowan model [10–15]. Functional connectivity in these models is typically measured using amplitude envelope correlations in MEG data, or in fMRI either by simulating the model on slow BOLD timescales [13,16], convolving model predictions using a hemodynamic response function [17–19], or incorporating a hemodynamic model (e.g., Balloon-Windkessel) [20–23].

Achieving realistic brain activity in biophysical models typically requires extremely fine tuning of parameters [24]. This contrasts with real brains, whose dynamics can be robust to quite large changes in white matter connectivity and grey matter structure, whether through learning, aging, development or disease [25]. There is increasing evidence that a fine balance between excitation and inhibition underpins a wide range of dynamics found in the brain [23,26–32]. However, how is this balance maintained when the brain is perturbed by changes that disrupt this balance? One possible explanation is that there are homeostatic mechanisms that continuously adjust brain networks to achieve the necessary balance. The balance between excitation and inhibition is often framed in terms of correlations in excitatory and inhibitory activity when analysing electrophysiological data, but it could equally be framed in terms of excitatory and inhibitory connection strengths at the connectome level.

Inhibitory synaptic plasticity (ISP), where the strength of local inhibitory connections changes depending on whether excitatory activity is above or below a target level of activity, is a parsimonious, biophysically plausible mechanism that directly modulates the balance between excitation and inhibition [33–36]. ISP can be readily implemented in simple biophysical models [27,37], and integration of ISP into a whole-brain neural mass model has been previously demonstrated with neural dynamics occurring on fMRI timescales [13]. However, the effects of ISP on electrophysiological timescales and the robustness of ISP regarding conductance delays are unexplored. Models on electrophysiological timescales have complex fast dynamics that could affect the outcome of plasticity on long timescales.

The aim of this study is to use a biophysical model to investigate whether ISP is successfully able to balance excitation and inhibition for models with ongoing oscillations on electrophysiological timescales, and to investigate the effect that ISP has on population-level dynamics and functional connectivity measured using MEG. For this purpose, we selected the well-established Wilson-Cowan neural mass model, which includes both excitatory and inhibitory populations and can be readily extended to incorporate ISP. By simulating neural activity on electrophysiological timescales and including conduction delays between brain regions, we investigate the effect of ISP on functional connectivity metrics typically used in MEG, using both amplitude envelope correlations and phase based measures of connectivity.

## Methods

### Ethics statement

All participants gave written informed consent and ethical approval was granted by the University of Nottingham Medical School Research Ethics Committee.

### Network model of neural activity

**Fig 1:**
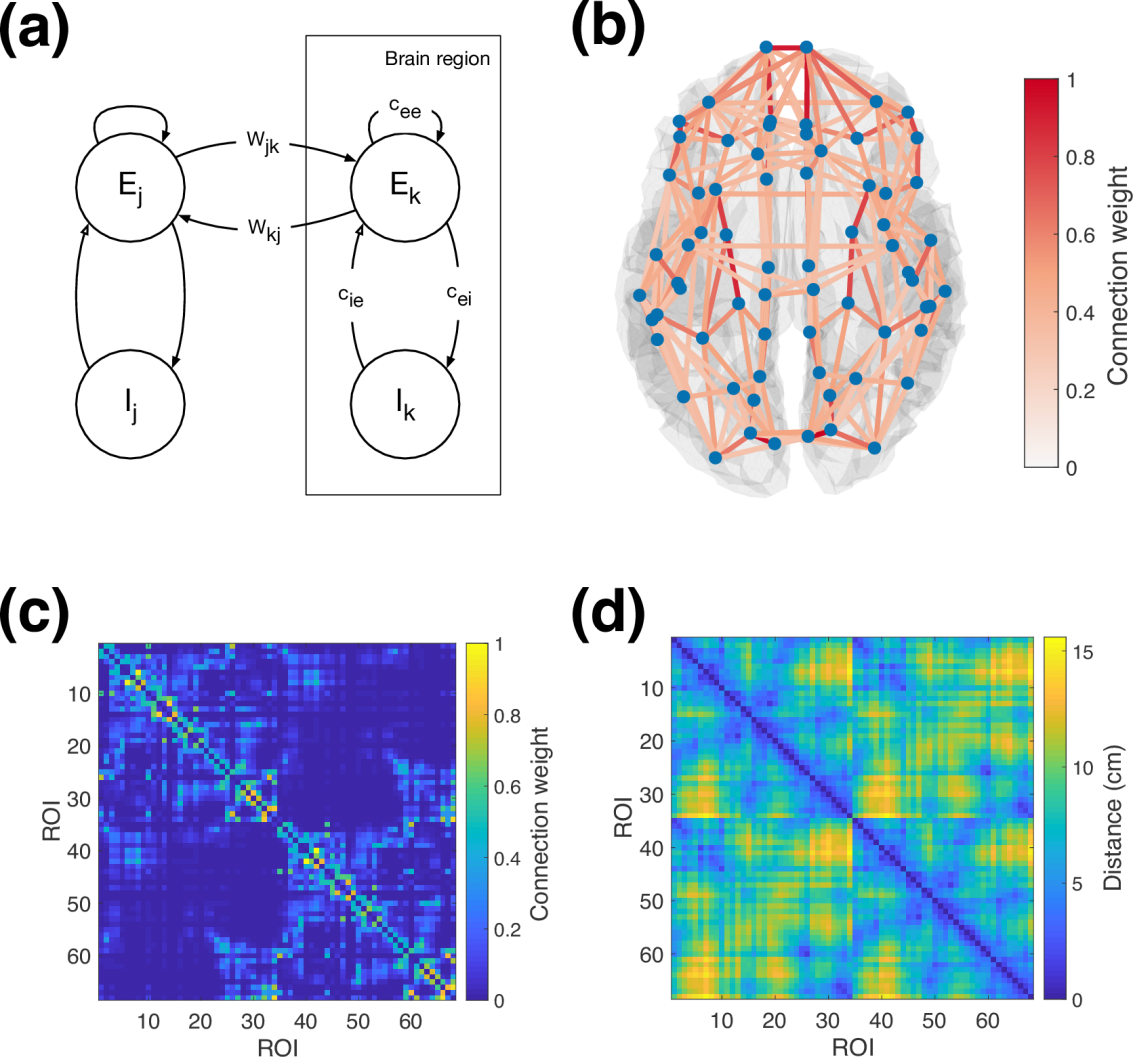
Overview of model structure. (a) Schematic of Wilson-Cowan neural populations and connections between, both within and between brain regions. (b) Overview of parcel centres and connectivity between them from the Desikan-Killiany parcellation and tractography. The strongest 10% of connections are displayed. (c) Anatomical connectivity matrix used for simulations (d) Distance matrix used for simulations. The labels mapping ROI index to brain region are provided in S1 Text.

We simulate neural activity using a network of neural masses, using the Wilson-Cowan model [10]. We divide the cortex into regions based on a grey matter parcellation, and model the dynamics in each brain region by an excitatory and inhibitory population of neurons (a unit), connected as shown in **Fig 1A**. Long-range white matter connections link excitatory populations across different brain regions with distance-dependent propagation delays. In accordance with previous work using this model [12,13,15], inhibitory connections are purely local, although long-range inhibition can be readily included in the future in the same way as long-range excitation.

Activity in the neural populations is governed by the equations [12,13]:

$$\tau_{e}\frac{dE_{k}\left(t\right)}{dt}=-E_{k}\left(t\right)+S\left(c_{ee}E_{k}\left(t\right)+c_{ie}^{k}\left(t\right)I_{k}\left(t\right)+P+\xi \left(t\right)+C\overset{n}{\underset{j=1}{\sum }}W_{jk}E_{j}\left(t-\tau_{jk}\right)\right),$$ (1)

$$\tau_{i}\frac{dI_{k}\left(t\right)}{dt}=-I_{k}\left(t\right)+S\left(c_{ei}E_{k}\left(t\right)+\xi \left(t\right)\right),$$ (2)

where *E*_*k*_ and *I*_*k*_ are the mean firing rates in the excitatory and inhibitory populations in brain region *k*, *τ*_*e*_ and *τ*_*i*_ are excitatory/inhibitory time constants, *c*_*ab*_ is the local connection strength from population *a* to population *b*, *P* is constant external excitatory input, and *ξ*(*t*) is a noisy input signal added into the dynamics. Long range white-matter connections *W*_*jk*_ from region *j* to region *k* incorporate a time delay *τ*_*jk*_, and are multiplied by a global coupling scaling *C*. Indices *k* and *j* range over the number of brain regions in the parcellation, *n*. The nonlinear response function *S* is a sigmoid function given by

$$S\left(x\right)=\frac{1}{1+e^{-\frac{x-\mu }{\sigma }}},$$ (3)

where *μ* and *σ* represent the mean firing threshold and variation in threshold for neurons in each population. Note that the numerator of Eq (3) corresponds to the maximum firing rate of the neural populations, so all firing rates here are represented as a non-dimensional fraction of the maximum firing rate. Actual firing rates could be simulated by multiplying the sigmoid by a dimensional constant, and accordingly dividing the connection strengths by the same constant. This changes the units of the state variables, but does not affect their dynamics with regard to the time constants, delays, or any of our measures of functional connectivity.

Without ISP, the local inhibitory coupling strength is constant and identical for all brain regions *k*, with $c_{ie}^{k}(t)=c_{ie}(0)$. To implement ISP, we use the framework developed by Vogels et al. [27] in which ISP is modelled as a spike-timing-dependent process dependent on both pre- and postsynaptic activity. The local inhibitory connection strength changes depending on the activity of the corresponding local excitatory population, according to [13,27]

$$\tau_{isp}\frac{dc_{ie}^{k}\left(t\right)}{dt}=I_{k}\left(t\right)\left(E_{k}\left(t\right)-\rho \right),$$ (4)

where *τ*_*isp*_ is the learning rate, and *ρ* is the target excitatory activity level, and the initial value $c_{ie}^{k}(0)=c_{ie}(0)$. As we are focused on slow plasticity where plasticity is decoupled from fast neural activity [38,39], the primary constraint on *τ*_*isp*_ is that it is large enough to ensure separation of timescales between Eqs (1 and 2) and Eq (4). Once *τ*_*isp*_ is sufficiently large, further increasing it will change how long the system needs to be simulated until plasticity ceases, but will not change the dynamics of the system once that point is reached. The fact that we only include long-range E-E connections results in an initial imbalance in the network which ISP acts against. In the real brain, the network would already be close to balanced, and ISP would instead serve to oppose more subtle changes, such as those introduced by other plasticity mechanisms.

### Model parameters

The parameter values used in this study are listed Table 1 and are based on those used in previous work by Deco et al. [12]. The parameters have been rescaled such that the maximum value of the sigmoid response is 1 and *μ* = 1 for simplicity, but this does not alter the dynamics of the system. The inhibitory time constant is larger than the excitatory time constant, consistent with previous studies [40,41] and cellular measurements in rodent [42]. For these parameters, an isolated unit transitions from a stable steady state to oscillations as the value of P increases beyond 0.34 (a Hopf bifurcation). The value of *P* at which the Hopf bifurcation occurs depends on the ratio *τ*_*e*_/*τ*_*i*_, while the frequency can be changed by rescaling both time constants in proportion. The parameters in Table 1 result in intrinsic oscillations at ~11 Hz, which is suitable for investigating functional connectivity in typical MEG frequency bands. In accordance with previous work [12], we set *P* = 0.31 such that the individual units are just below their oscillatory threshold, and large oscillations in the network arise only when long-range coupling is included. We set the nominal ISP target level of activity to *ρ* = 0.15, which is a relatively low level of activity that corresponds to an isolated unit being in an oscillatory state.

**Table 1:** Model local parameter values. Parameters are based on previous work by Deco et al. [12].

Parameter	Description	Value	Units
*c*_*ee*_	Local excitatory to excitatory coupling	3.5	-
*c*_*ei*_	Local excitatory to inhibitory coupling	3.75	-
*c*_*ie*_(0)	Initial local inhibitory to excitatory coupling	-2.5	-
μ	Firing response threshold	1	-
σ	Firing threshold variability	0.25	-
*P*	Constant excitatory input	0.31	-
*τ*_*e*_	Excitatory time constant	0.01	s
*τ*_*i*_	Inhibitory time constant	0.02	s
ρ	Nominal ISP target firing rate	0.15	-

### Structural connectivity

In this study, we simulated a cortical brain network using the Desikan-Killiany parcellation [43] with 68 brain cortical regions covering the brain cortex bilaterally. Structural connectivity weights between these regions were estimated using diffusion MRI probabilistic tractography and data from the Human Connectome Project [44–46]. Briefly, fibre orientations were estimated from distortion-corrected data [47] using a model-based spherical deconvolution approach, as implemented in FSL [48,49]. Up to three fibre orientations were detected per white matter voxel and were used for probabilistic tractography, which was performed using FSL’s probtrackx2 (https://fsl.fmrib.ox.ac.uk/fsl/fslwiki/FDT) [50]. The white/grey matter boundary surface was used as a seed, since this reduces biases observed using whole-brain seeding [51,52]. Streamlines were seeded from *N* = 60,000 standard-space vertices [46] on the boundary surface (10,000 streamlines per seed). Anatomical constraints were imposed to reduce false positives. Specifically, we allowed streamlines to hit the white matter/grey matter boundary not more than twice, and also streamlines were allowed to enter subcortical volumes, propagate within them, but terminate upon exit, as suggested in [53]. The pial surface was further used as a termination mask to ensure estimated paths do not “jump” between neighbouring gyri. The number of streamlines reaching each vertex in the WM/GM boundary was recorded, and this was normalised by the total number of valid streamlines propagated, giving a dense NxN “connectivity” matrix. Using the cortical parcellation, this matrix was reduced to a 68 x 68 parcellated connectivity matrix, by computing for each pair of regions the mean connectivity between all pairs of vertices they were comprised of. Forty subjects were processed and their resulting connectivity matrices were averaged. The connection matrix was log-transformed to account for algorithmic bias in the tractography [54], and normalised by dividing by the largest value, yielding the structural connectivity used in the simulations shown in **Fig 1B and 1C**. Propagation delays between brain regions were approximated using the barycentric distances between regions (shown in **Fig 1D)**, with a uniform conduction velocity that was varied as one of the model parameters.

### Numerical integration

To generate model predictions comparable to MEG, we integrated the system with a sufficiently small time step (Δ*t* = 1 × 10^−4^ s) using a 4th order Runge-Kutta scheme for 500 seconds. The noise signal *ξ*(*t*) was sampled from a Gaussian distribution (zero mean, standard deviation 0.01) in each population at each time step, and was interpolated for the intermediate steps in the integration (noting that this means our noise input is not white noise). The initial conditions for the system were randomized, and the first 15 seconds of the simulation were discarded to remove transient effects due to the initial conditions. The simulated output was downsampled to 300Hz to reduce storage requirements for comparison to typical MEG data.

In accordance with previous studies, we include a small amount of noise to improve the robustness of the simulation [15]. Without noise, we find that the system is prone to becoming trapped in highly periodic oscillatory states that are not physiologically realistic. However, these states are only marginally stable, such that even a very small amount of noise is sufficient for the system to escape the attractor. Thus these states are also not biologically plausible, because the ubiquitous external perturbations and sources of noise in the brain, e.g. thermal noise, synaptic transmission failures, would prevent the system from remaining in such a state [55,56]. We select the noise amplitude such that it is much smaller than the nonlinear oscillations that drive functional connectivity in the model. Our results therefore primarily reflect the nonlinear interactions between populations rather than noise, and our results are qualitatively robust to moderate variation of the step size and noise amplitude (as shown in **Fig A2** in S1 Supplementary Material).

To simulate ISP, we integrated the system for 1500 seconds. To improve computational tractability, we accelerate ISP in the initial portion of the simulation. For the first 500 seconds we set *τ*_*isp*_ = 2.5s, the next 500 seconds *τ*_*isp*_ = 10s, and the final 500 seconds *τ*_*isp*_ = 20s. This procedure strikes a balance between ensuring that ISP converges in a computationally feasible amount of time, while also ensuring that the timescale differences are sufficient to decouple the neural oscillations from ISP. At the end of this period, ISP was disabled, and the simulation run for a further 500 seconds. As we do not expect changes in synaptic strength to drive fast neuronal dynamics over short periods of time and are focused on the dynamics of the balanced networks that are the end result of ISP, we turned off ISP for the final run to ensure that our measures of activity and stationary functional connectivity are not affected by ongoing changes in synaptic strength [13].

### Synchrony

Synchrony and variability in synchrony (metastability) are common global metrics of activity for coupled oscillator models. In particular, variability in synchrony is associated with realistic brain activity [1,57,58]. The synchrony order parameter *R*(*t*) is obtained by summing over the analytic phase of each oscillator in the network [1,4,57]

$$R\left(t\right)e^{i\theta \left(t\right)}=\frac{1}{N}\overset{N}{\underset{k=1}{\sum }}e^{i\phi_{k}\left(t\right)}$$ (5)

This order parameter is 1 if the oscillators are completely synchronized (their phases are the same), and 0 if their phases are uniformly randomly distributed.

For phase oscillators such as the Kuramoto model, the phase of the oscillator is well defined because it is simply the state variable of the model. In contrast, for the Wilson-Cowan model it is necessary to use a technique like the Hilbert transform to estimate phase. The analytic signal is derived from the original signal using

$$X_{a}(t)=X(t)+iH(t)$$

where *X*_*a*_ is the analytic signal, *X* is the original signal, and *H* is the Hilbert transform of *X*. Writing this in polar form gives

$$X_{a}(t)=A(t)e^{i\phi (t)}$$

where *A*(*t*) is the amplitude envelope timecourse, and *ϕ*(*t*) is the phase timecourse. This phase is only readily interpretable for narrowband signals, which means the oscillatory activity needs to be filtered prior to estimating synchrony. We used a 4^th^ order, two pass Butterworth filter implemented by FieldTrip [59].

### Data analysis

We used eyes open resting state MEG data from 55 healthy controls (ages 18–48, mean age 26.5, 35 males), acquired at the University of Nottingham as part of the UK MEG Partnership. Data were acquired with a 275-channel axial gradiometer CTF MEG system (MISL, Conquitlam, Canada) at a sampling rate of 1200 Hz, and downsampled to 600Hz with a 300 Hz low pass anti-aliasing filter. Synthetic third order gradiometer correction was applied to reduce external interference. Subjects were seated in the scanner and presented with a fixation target while 300 seconds of data were recorded. Head position was continuously tracked using three head position indicator (HPI) coils, placed at the nasion and left and right preauricular points. Structural MRI scans for each subject were acquired at 0.8mm isotropic resolution using a Philips 7T Achieva MRI scanner running a phase-sensitive inversion recovery sequence [60]. To coregister the MEG system geometry to the subject’s anatomical MRI, a Polhemus FASTRAK 3D digitiser system was used to record the position of the fiducial points and the subject’s head shape. The locations of the MEG sensors relative to individual brain anatomy were determined by registering the digitized head surface to the structural MRI. The structural MRI was registered to the MNI152 standard brain and all source space analysis was then performed in MNI space.

For analysis, the data were imported into SPM12 format, and downsampled to 250 Hz using an anti-aliasing low-pass filter. High-pass filtering (cut-off 0.1 Hz) and a notch filter were subsequently applied to attenuate slow drifts and line noise. For outlier detection, resting state data were epoched into pseudo-trials of 2s length, and the signal standard deviation was estimated once per trial. Subsequently these estimates were subject to a robust fit using the bisquare distribution. Extreme trials were identified by a regression coefficient smaller than 0.05 and were discarded. The data were visually inspected for remaining artefacts. The data were bandpass filtered from 1-45Hz, and then beamformed onto an 8mm grid [61]. For each brain region, a single activity timecourse was computed as the first principal component of the activity in voxels belonging to that region [62].

To compensate for spatial leakage (which can affect certain connectivity metrics), we use the symmetric multivariate orthogonalisation algorithm developed by Colclough et al. [62]. In summary, this multivariate algorithm removes all zero-lag correlations between all parcel timecourses simultaneously, by projecting them onto a new orthogonal basis. We also apply the orthogonalisation procedure to the model when computing connectivity metrics that require leakage correction in MEG data, to ensure that the underlying activity in the model is consistent with experimental data when processed through the same analysis pipeline. That is, the model should generate predictions comparable to experimental data when zero lag correlations are removed, even though there is no spatial leakage in the model, because genuine zero lag correlations have also been removed in the data.

### Functional connectivity analysis

We analyse static functional connectivity in the data and in the model using three metrics–amplitude envelope correlation (AEC), phase locking value (PLV), and phase lag index (PLI) [63]. Of these, AEC and PLV are affected by spatial leakage and require signal orthogonalisation, while PLI is insensitive to spatial leakage and does not require orthogonalisation. We include PLI in addition to AEC and PLV to ensure the model does not rely on the orthogonalisation procedure to produce connectivity similar to experimental data.

To compute the amplitude envelope correlation, analytic signal corresponding to the orthogonalised parcel timecourses is computed, and then the amplitude envelope component is extracted and downsampled to 1Hz [64–67]. The connectivity matrix is obtained by computing the Pearson correlation between every pair of the downsampled envelope timecourses. AEC requires orthogonalisation because additive mixing of the parcel timecourses due to spatial leakage introduces zero-lag correlations in the raw signals that consequently result in correlations in the envelope timecourses. Removing all zero-lag correlations implies that any remaining envelope correlations are due to mechanisms other than spatial leakage.

The phase locking value [68] is calculated from the phase component of the analytic signal, again computed using the orthogonalised parcel timecourses [63]. PLV is a pairwise measure of synchronization between two signals, given by

$$PLV_{ij}=|\langle e^{i{(\phi }_{i}(t)-\phi_{j}(t)}\rangle |$$

If the phase difference is constant over time, then the PLV will approach 1, whereas if the phases of the two signals are random relative to each other, then the PLV will be 0. PLV requires orthogonalisation because additive mixing of the parcel timecourses introduces a constant phase relationship between them, artificially increasing the PLV.

The phase lag index [62,69] aims to quantify asymmetry in the distribution of the phase difference between two signals. It is calculated from the phase component of the analytic signal using the raw (non-orthogonalized) parcel timecourses:

$$PLI_{ij}=|\langle \mathrm{sign}(\sin{(\phi }_{i}(t)-\phi_{j}(t))\rangle |$$

PLI does not require signal orthogonalization because while spatial leakage decreases the phase difference between the signals, it does not make the distribution of phase differences less symmetric.

**Fig 2:**
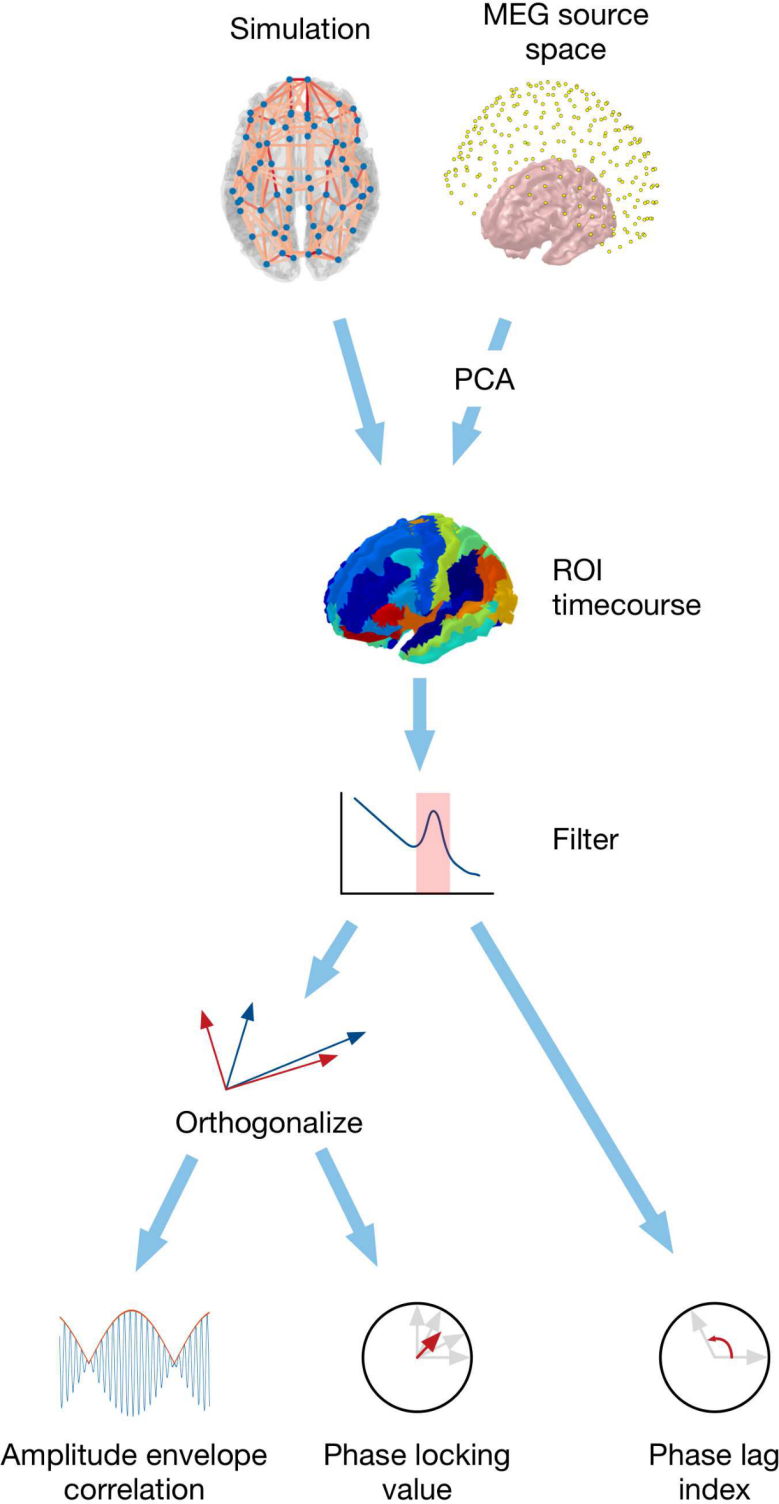
Overview of analysis pipeline for simulated neural activity and MEG data. After computing activity timecourses for each region, both simulated and real signals are processed through the same pipeline.

The overall analysis pipeline from simulated or measured data to connectivity estimates is shown in Fig 2. As excitatory pyramidal cells contribute most strongly to EEG/MEG signals, we associate activity in the excitatory populations of the model with signals in experimental data [70–72], although future work may wish to model contributions from both excitatory and inhibitory populations by modelling postsynaptic currents in more detail.

## Results

**Fig 3:**
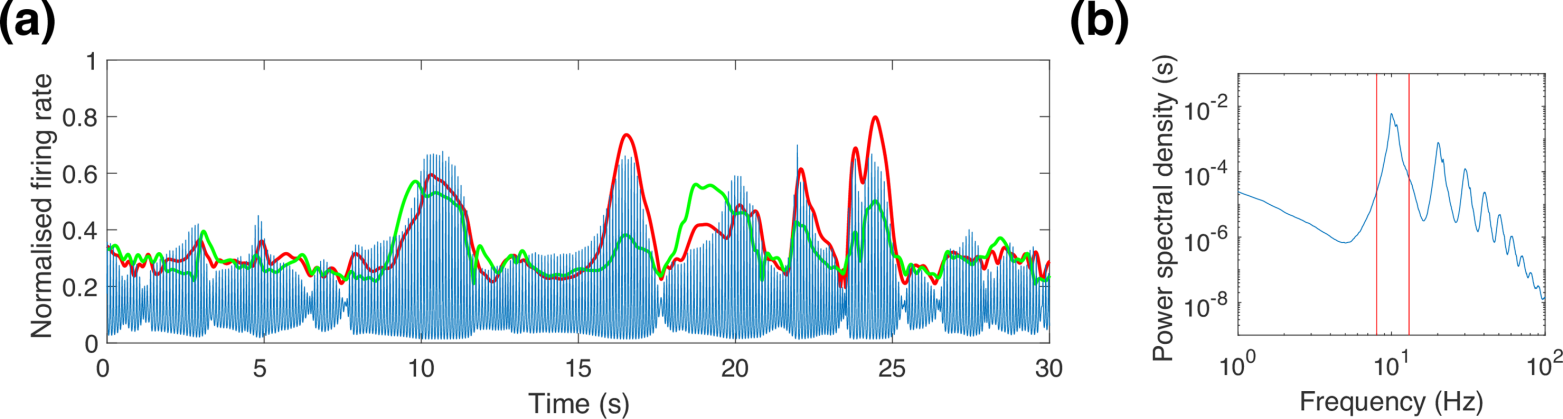
Typical model neural activity. (a) Raw excitatory activity timecourse (blue) and corresponding orthogonalised alpha band envelope (red) for the left pericalcarine parcel, which is well correlated with the right pericalcarine as shown in Fig 5B. The envelope timecourse in right pericalcarine (green) is strongly correlated with the envelope timecourse in left pericalcarine–these correlations correspond to the AEC reported in Figs 4 and 5. (b) Power spectrum of excitatory activity, averaged over all brain regions. The red bars show the position and size of the alpha band analysis window relative to the spectral peak.

In our MEG data, the strongest peak in the spectrum lies in the alpha band, as does the strongest functional connectivity (largest mean AEC). As exemplified in **Fig 3**, the predicted activity in the model is highly oscillatory, and is dominated by the fundamental oscillation in the alpha band, with harmonics extending to higher frequencies. The functional connectivity patterns are very similar for each harmonic peak (as shown in **Fig A1** in S1 Supplementary Material), so we focus only on the fundamental oscillation in the alpha band (8-13Hz). This is consistent with other similar networks of neural masses that generally do not predict band specific connectivity patterns [8].

### Functional connectivity similarity

**Fig 4:**
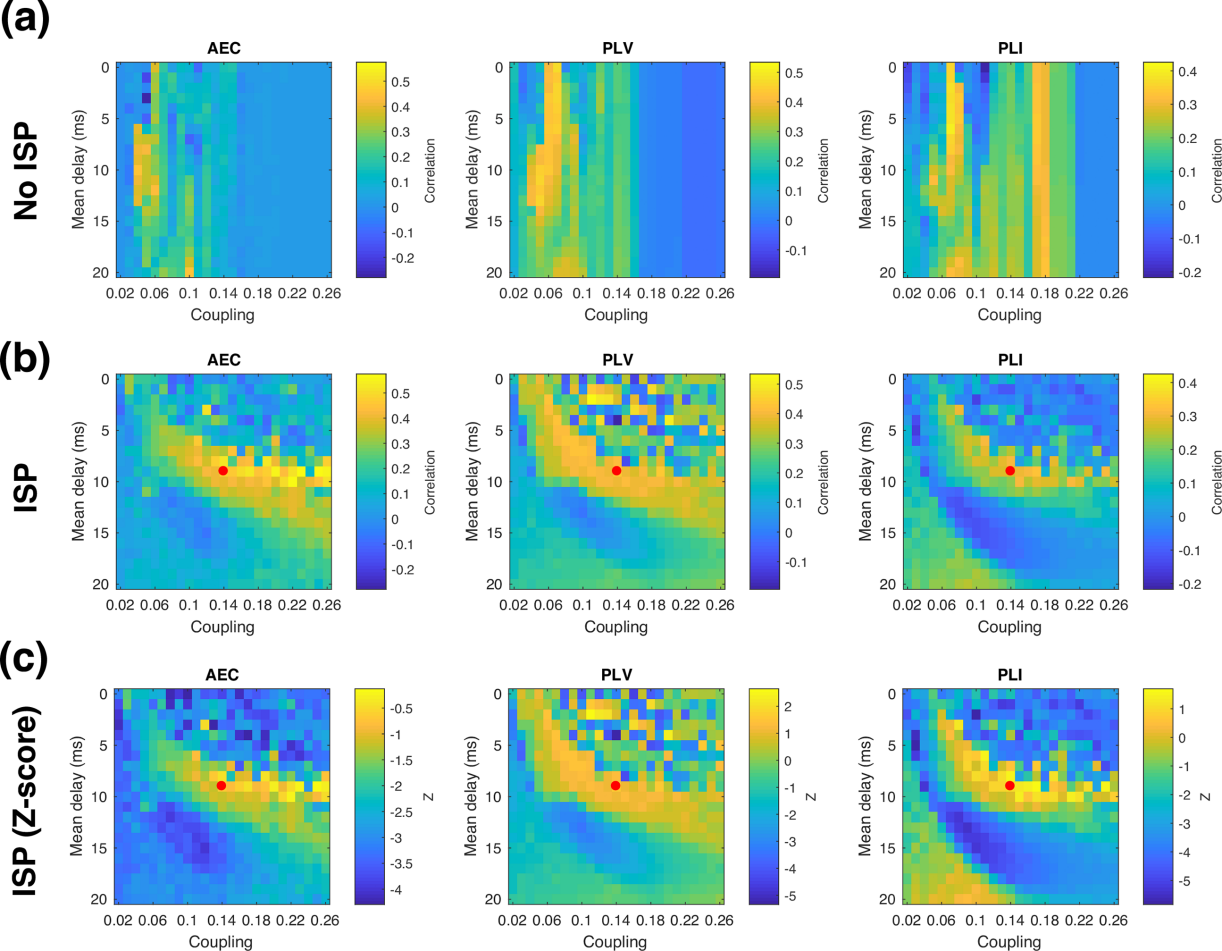
Similarity in functional connectivity metrics. Similarity between model and data in each functional connectivity metric is shown for different global coupling and velocity regimes (a) Without ISP, (b) With ISP. The red dot marks the representative parameters from the optimal regime shown in **Figs 5** and **7**. (c). Similarity measure with ISP expressed as a z-score based on individual variability.

To assess the influence of ISP in the model behaviour, we quantified similarity between connectivity matrices by computing the correlation coefficient for the upper triangular part of each (symmetric) connectivity matrix for different scenarios. As shown in **Fig 4A**, without ISP the model exhibits high similarity with real group average AEC and PLV over a narrow range of coupling strengths, and with intermediate delays (5-15ms). The functional connectivity estimated using PLI is acceptable, but not optimal in this same regime–instead, the best fit with experimental data is obtained at somewhat shorter delays, and slightly stronger coupling strengths.

With the inclusion of ISP, the model can reproduce alpha band functional connectivity over a wide range of global coupling strengths, as shown in **Fig 4B**. We find that the range of delays over which realistic functional connectivity is exhibited is relatively unchanged by the addition of ISP (when collapsed over coupling strength), while sensitivity to long-range coupling is greatly reduced, as expected. Notably, in the model with ISP, the optimal parameter regime for matching experimental data is the same for all three functional connectivity metrics. This demonstrates that it is possible to select a single operating point that matches functional connectivity in both amplitude and phase connectivity measures, and regardless of whether spatial leakage correction is applied or not.

### Comparison with individual variability

**Fig 5:**
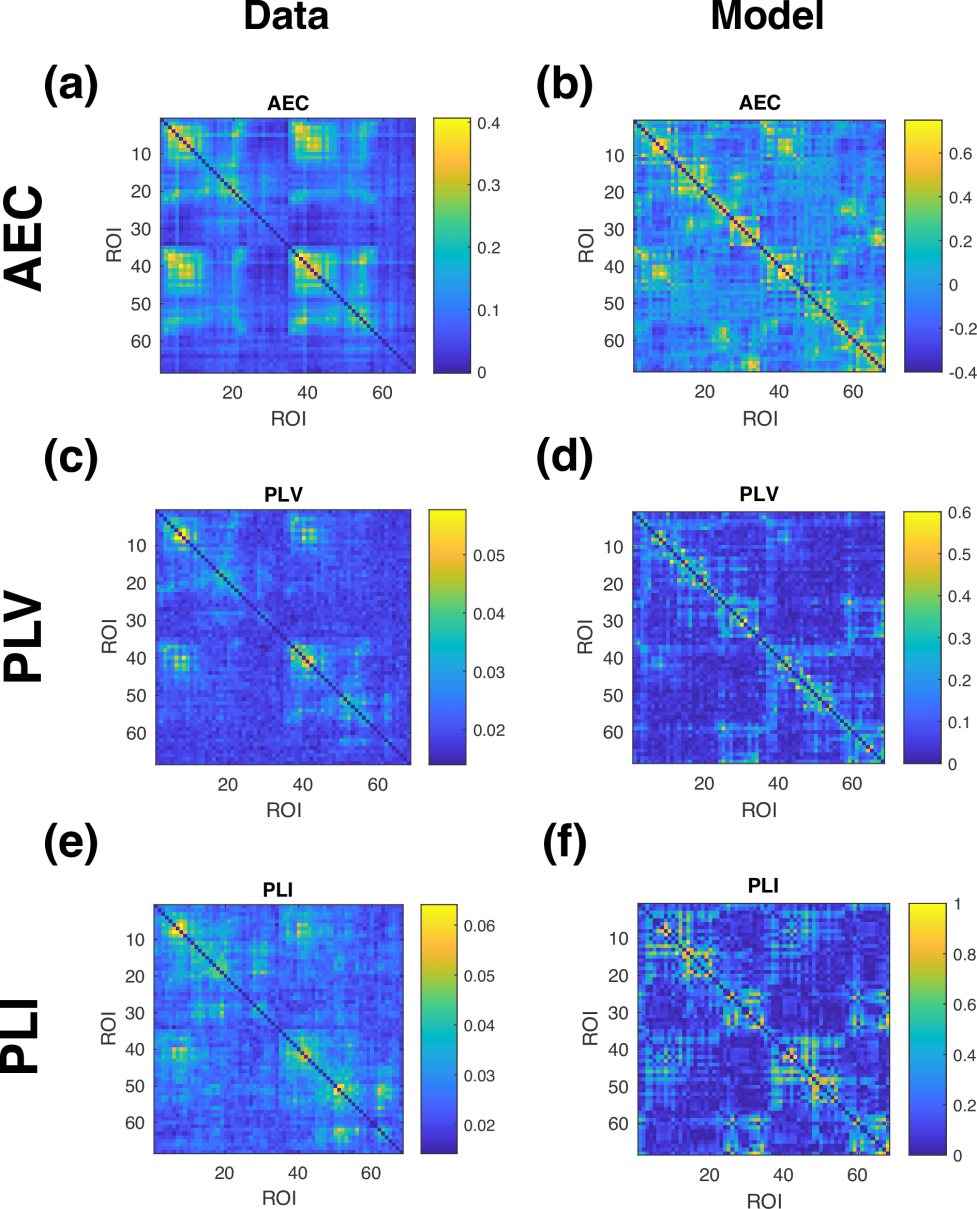
Alpha band functional connectivity profiles in data and model. Functional connectivity in (a,b) AEC, (c,d) PLV,and (e,f) PLI, shown in data, and in the model for the parameters marked by the red dot in **Figs 4, 6** and **7**.

The extent to which the model can predict real group average functional connectivity is limited by the fact that we use an average structural connectivity matrix, rather than averaging functional connectivity over individual connectomes (notwithstanding the uncertainties in estimation of the connectomes themselves). However, the magnitude of the similarity between the model and data shown in Fig 4A and 4B is difficult to interpret without a point of comparison. The correlation between the matrices shown in Fig 5 is 0.48 for AEC, 0.43 for PLV, and 0.28 for PLI–does this indicate that the model is performing well? Given that we are comparing functional connectivity estimated from a single connectome to the group average, a suitable point of comparison is the typical level of similarity observed between real individual’s functional connectivity profile and the real group average. To estimate this, we computed the correlation between each real subject’s functional connectivity and the real group average connectivity computed with that subject left out. The similarity in connectivity between real individuals and the real group average was 0.60 ± 0.17 for AEC, 0.29 ± 0.09 for PLV, and 0.22 ± 0.06 for PLI. We can then test whether the model’s performance is significantly different by computing the difference between model similarity and mean individual similarity, and then dividing by the standard deviation of the individual similarity. This converts the model similarity to a Z-score based on individual variability, shown in **Fig 4C**. The null hypothesis that the model’s similarity comes from the same distribution is then rejected if |*Z*| > 1.96. For the optimal parameter regime, |*Z*| < 1.96 in all three connectivity measures, which indicates that our observed model performance is not significantly different to the typical similarity observed in real individuals.

### Functional connectivity profiles

The functional connectivity profiles for a representative set of parameters in the optimal parameter regime (the red dot in **Fig 4**) are shown in **Fig 5**. In general, the model predicts stronger functional connectivity than is seen in the group average data, although we note that real individual functional connectivity can also be considerably stronger than the group average. To verify that this is not a consequence of the low level of noise included in the simulation, but is an intrinsic property of the nonlinear dynamics of the system, we repeated the simulation shown in **Fig 5** for a range of different noise amplitudes (as shown in **Fig A2** in S1 Supplementary Material). We find that increasing the noise by a factor of 10 provides qualitatively similar results, including the magnitude of the connectivity metrics. This robustness partly reflects the fact that the response time of the system limits the effect of the noise, acting as a low-pass filter that averages out the noise over short periods of time.

### Synchrony and metastability

**Fig 6:**
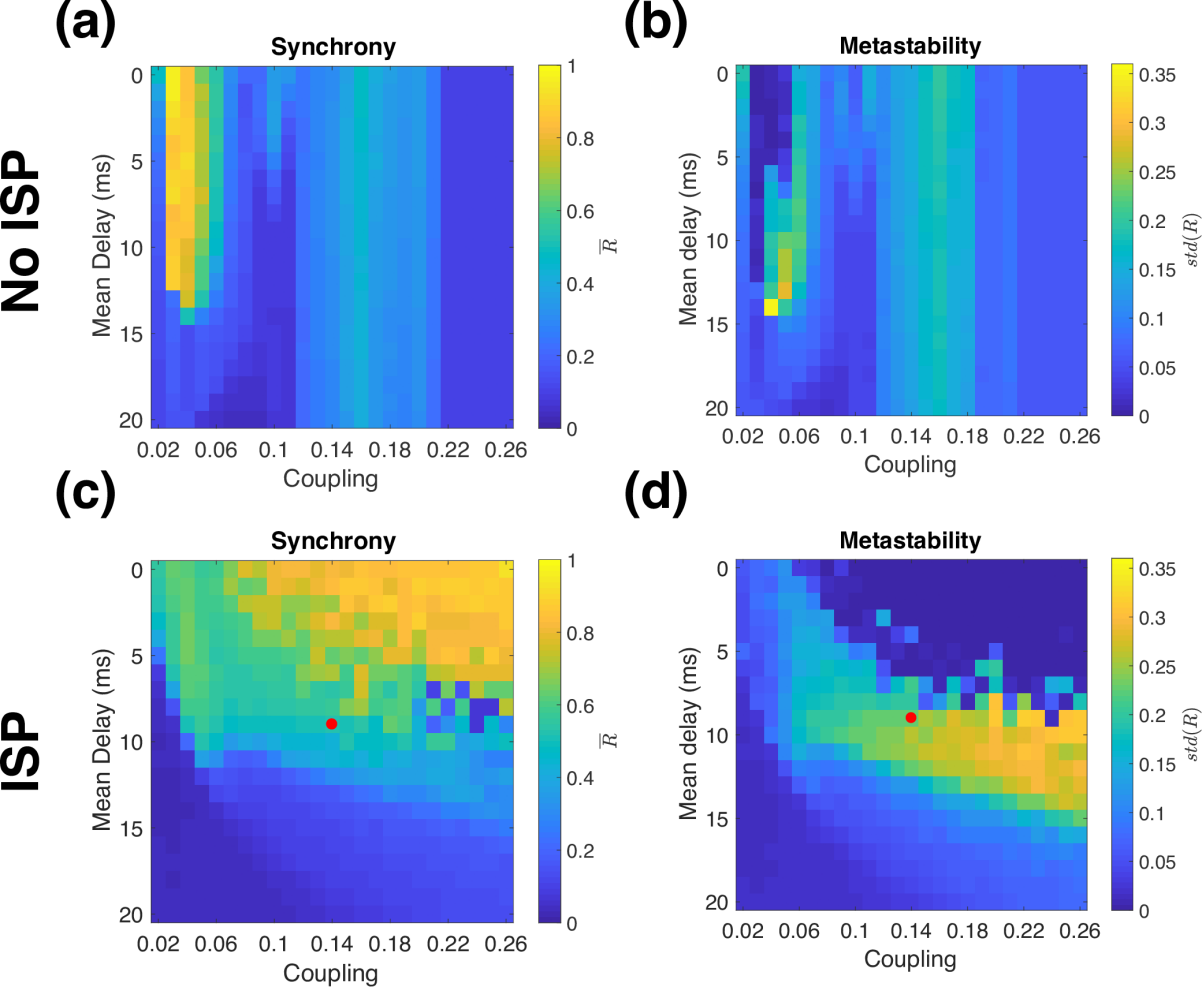
Synchrony and metastability. Alpha band synchrony (averaged over time) and metastability (standard deviation of synchrony), in the model (a,b) without ISP (c,d) with ISP. The red dot corresponds to the parameters shown in **Figs 5** and **7**.

The time-averaged synchrony (mean value of *R*(*t*) as defined in **Eq 5**) and metastability (standard deviation of *R*(*t*)) are shown in **Fig 6**. Compared to previous work using coupled Kuramoto phase oscillators [1], the Wilson-Cowan model is more sensitive to network coupling strength because the amplitude of oscillations in neural activity can vary. Networks of Kuramoto oscillators exhibit highly synchronized oscillations when strongly coupled, but this is not the case for the Wilson-Cowan model, as shown in **Fig 6A** and **6B.** Without ISP, the model exhibits high synchrony over a narrow range of couplings, but a wide range of delays. As coupling becomes stronger, synchrony drops markedly. As coupling strength is increased further, the global synchrony rises again. This occurs because strong coupling causes the most strongly connected neural populations to enter a high-activity stable steady state. In this regime, it is difficult to interpret the global synchrony because different brain regions exhibit qualitatively different dynamics. When coupling increases further (for our parameters, when global coupling is stronger than 0.21), all the units converge to a high activity steady state with noise-driven fluctuations in activity and low synchrony. However, when excitation and inhibition are locally balanced, the global network dynamics become much more similar to those seen with simple phase oscillators. We recover the same regime of high synchrony occurring at progressively longer delays as coupling strength increases, as well as the existence of a metastable regime with high variability in synchrony at the interface between the high and low synchrony regimes of the model (**Fig 6C** and **6D**).

While synchrony and metastability are useful to analyse the model dynamics, they are not commonly used to study MEG data because they are extremely coarse measures that aggregate activity over the entire brain at each time point. As these measures are susceptible to spatial leakage, comparison to data must include a leakage correction step. We show synchrony and metastability in the model including orthogonalisation in **Fig A3** in S1 Supplementary Material. In summary, in the optimal parameter regime of the model, the predicted synchrony and metastability are both larger than in data by a factor of 2–3, although the effect of measurement noise in the data is not accounted for in the model.

### E/I balance

**Fig 7:**
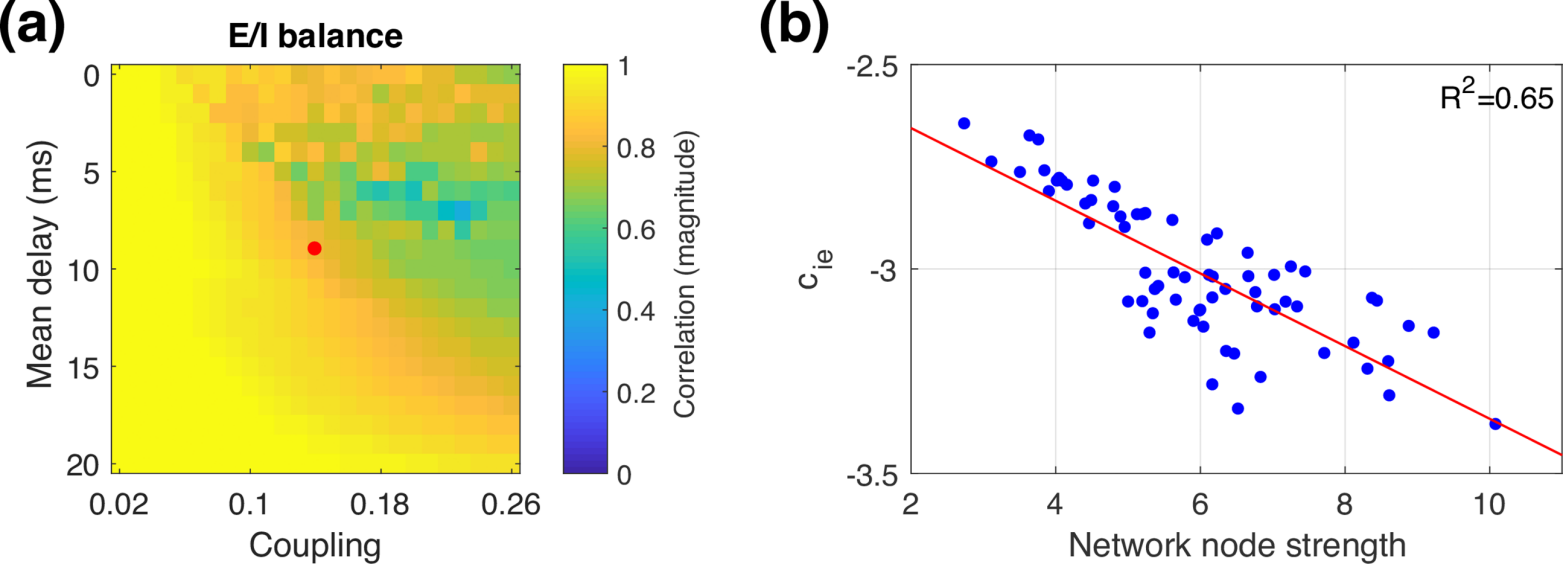
Balance of excitation and inhibition. (a) Magnitude of correlation between node strength and ***c***_***ie***_ for a range of global couplings and delays. The red dot corresponds to the parameters shown in **Fig 5**. (b) ***c***_***ie***_ values plotted against network node strength, for the parameters marked in panel (a) by the red dot, with a linear fit (red line).

We hypothesised that ISP would serve as a mechanism to balance excitation and inhibition in the network. This balance is inherently disrupted because we only include long-range excitatory connections. Balancing excitation and inhibition therefore requires that inhibition is increased more for brain regions that have stronger long-range couplings. To quantify whether such a balance has been achieved, we can examine the correlation between the node strength (row-sum of the structural connectivity matrix, which corresponds to the sum of long-range excitatory inputs) for each brain region, and the local inhibitory synaptic strength after ISP. Note that without ISP, the inhibitory synaptic strengths are independent of node strength and they are therefore uncorrelated. As shown in **Fig 7A**, for most delay/coupling parameter combinations, ISP successfully balances excitation and inhibition, with a strong correlation between long range excitation and local inhibition. The relationship is particularly clear in the low synchrony regime, either with weak coupling or large delays. In the high synchrony regime, ISP is somewhat less successful at balancing excitation and inhibition, and the E/I balance shown in **Fig 7A** becomes a snapshot of ongoing changes in inhibition (as shown in **Fig A5** in S1 Supplementary Material). **Fig 7B** shows the clear relationship between excitation and inhibition in the metastable regime, where realistic functional connectivity is achieved.

### Different ISP targets

To investigate the sensitivity of our findings to the choice of target activity level *ρ*, we repeated our simulations for *ρ* = 0.10 and *ρ* = 0.30 corresponding to a low-activity target and a high-activity target, respectively. For an isolated unit, at the Hopf bifurcation the mean excitatory activity level is 0.12, which means that a target of *ρ* = 0.10 is low enough that for low coupling values, the units can be in a low-activity, noise-driven steady state rather than undergoing nonlinear oscillations. In contrast, all tested parameters provide nonlinear oscillations for *ρ* = 0.15 and *ρ* = 0.30.

**Fig 8:**
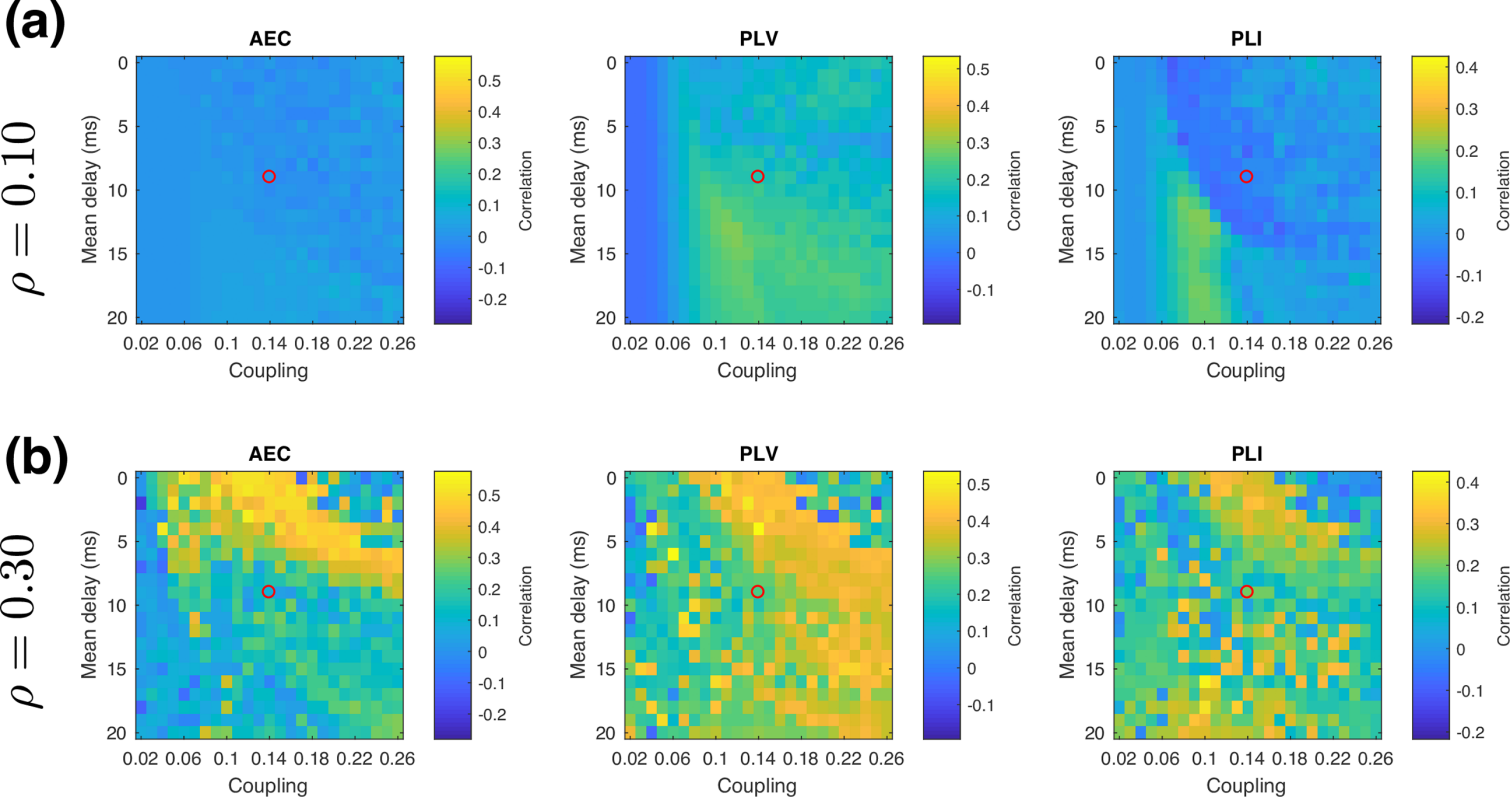
Different ISP target activity levels. Similarity between model and data is shown for each functional connectivity metrics for (a) low and (b) high ISP targets. The open circles correspond to the representative optimal delay and coupling values shown in **Fig 4** and used in **Figs 5** and **7**.

As shown in **Fig 8**, with *ρ* = 0.10 we did not find realistic functional connectivity for any of the tested delays and couplings. The low ISP target gives rise to two distinct regimes (as shown in **Fig A4** in S1 Supplementary Material)–a stochastic regime at low coupling strength, and a synchronous oscillatory regime at high coupling strength. In the stochastic regime, realistic functional connectivity can be produced when the model is close to instability, so that some of the eigenmodes of the system are weakly damped and thus their corresponding connectivity profiles become visible against the noise. In contrast to previous work [21], the stochastic regime does not give rise to realistic functional connectivity in the present study, suggesting that ISP has moved the system too far from instability. At higher coupling strengths, the oscillatory regime gives rise to stronger functional connectivity, but the orthogonalisation procedure eliminates most of the AEC because the raw signals are unrealistically highly correlated. As a result, neither regime gives rise to realistic functional connectivity.

On the other hand, with *ρ* = 0.30 our findings are qualitatively similar, with a parameter regime that gives rise to realistic functional connectivity at increasingly long delays as coupling strength increases. The magnitude of similarity between model and data is also comparable. However, the optimal parameter regime is shifted to shorter delays for the same coupling strengths. Notably, with *ρ* = 0.30 it is possible to obtain realistic functional connectivity even with no delays in the network, which is not possible with *ρ* = 0.15.

### Homogeneity vs heterogeneity

**Fig 9:**
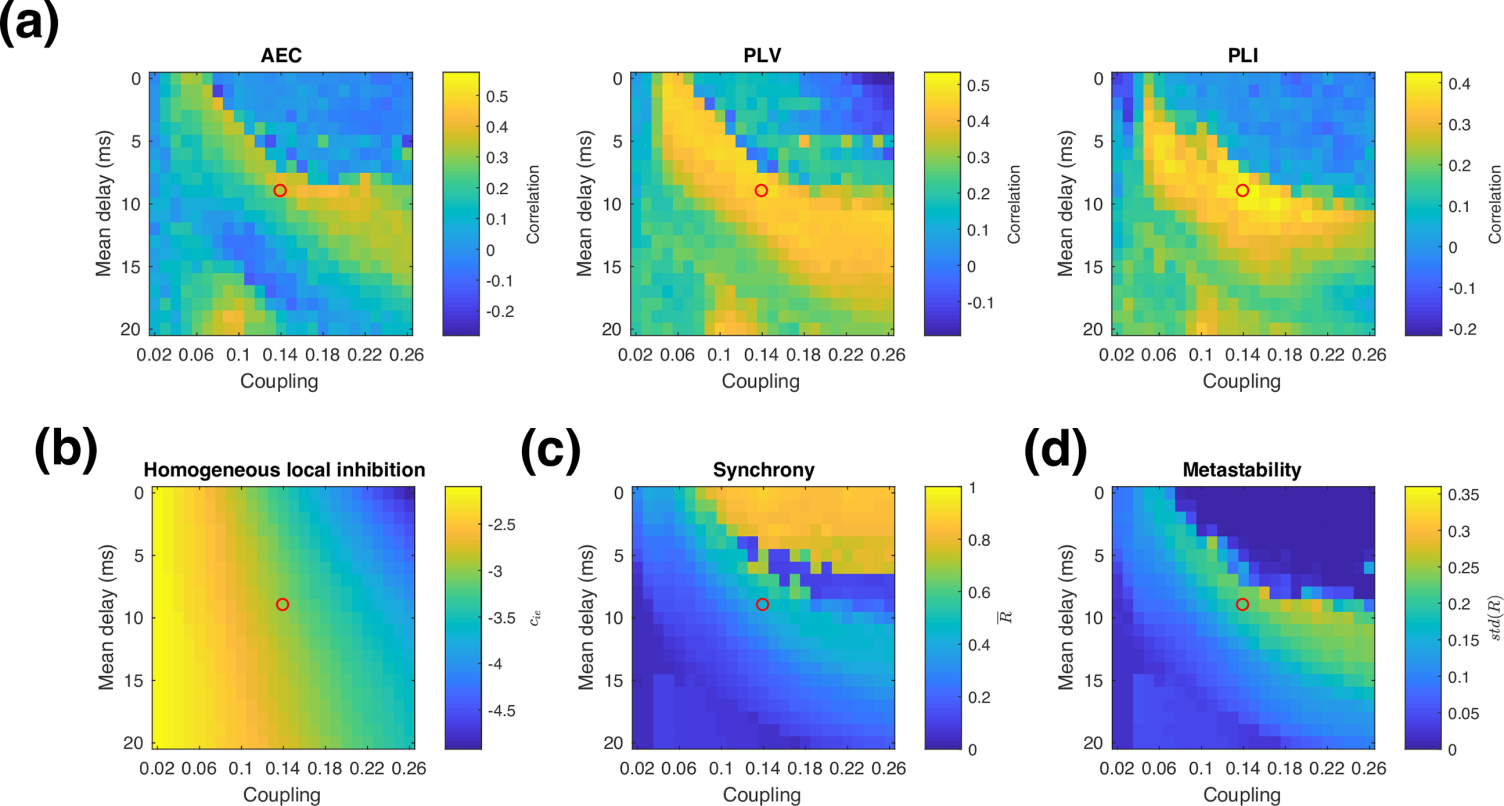
**Homogeneous model parameters**. (a) Functional connectivity similarity between model and data with uniform ***c***_***ie***_ set to the mean value of ***c***_***ie***_ for the corresponding ISP simulation at the same global coupling and delay values (b) Mean value of ***c***_***ie***_ obtained from ISP simulations (c) alpha band synchrony (averaged over time), and (d) metastability (standard deviation of synchrony). The open circles correspond to the representative optimal delay and coupling values shown in **Fig 4** and used in **Figs 5** and **7**.

Finally, we assessed the importance of locally balancing excitation and inhibition within every brain region, by testing the effect of coarsely balancing excitation and inhibition at the global level without introducing heterogeneity in the model parameters. We approximated this global balance by using the results of the ISP simulations–for each global coupling and delay, we computed the spatial average of *c*_*ie*_ after ISP, and then used this average within every brain region. In effect, this is an offline optimization. **Fig 9A** shows the resulting similarity in functional connectivity measures, and **Fig 9B** shows the homogeneous value of *c*_*ie*_ used for each delay and coupling value tested. As with the fine local balancing of excitation and inhibition in **Fig 4B**, sensitivity to global coupling is greatly reduced compared to **Fig 4A** where there is no regulation of inhibition. For PLI and PLV, the coarsely balanced simulations reach a maximum correlation between model and data that is similar to the locally balanced simulations, while for AEC performance of the coarsely balanced simulations is somewhat lower. We also examined the synchrony and metastability for these simulations, shown in Fig 9C and 9D. Comparing these to Fig 6C and 6D, we find qualitatively similar regimes of high synchrony, low, synchrony, metastability, and realistic functional connectivity as in the locally balanced ISP case.

### ISP convergence

For some points in parameter space, there are ongoing changes in inhibitory synaptic strength, indicating that the homeostatic mechanism is unable to converge. If ISP fails to converge, this indicates there are still sizable discrepancies between the target activity level and the actual activity level, which drives ongoing plasticity. We can quantify this by computing the standard deviation of *c*_*ie*_, after providing enough time for the system to converge first, if indeed convergence is possible. Convergence of ISP is examined in detail in the supplementary material, as shown in **Figs A5** and **A6** in S1 Supplementary Material. In summary, convergence tends to be robust for parameters that give rise to realistic neural activity, but fails in the high-synchrony regime, and in the low-synchrony regime depending on the ISP target.

## Discussion

We have used a large-scale biophysical neural mass model with local inhibitory synaptic plasticity (ISP) to investigate the effect of dynamic homeostatic balancing of excitation and inhibition on network synchronization and functional connectivity. Our main results are:

1. *ISP successfully regulates local activity on MEG timescales*. Online balancing of excitation and inhibition is possible both with ISP and fast oscillatory neural dynamics. Online balancing has been previously demonstrated on fMRI timescales [13], but is potentially complicated on MEG timescales due to the large nonlinear oscillations. ISP is a biophysically plausible, online way to implement the types of offline optimization of inhibition, such as feedback inhibition control (FIC) [23], that have been shown in previous work to promote realistic neural dynamics. Building on previous work [13], we also investigated the effect of changing the propagation velocity in the network, and found that functional connectivity and ISP convergence both perform well for realistic delays.
2. *ISP results in more realistic functional connectivity*. As expected, without ISP the model predictions are highly sensitive to coupling strength. When ISP is included, the sensitivity to coupling strength is reduced, making it possible to obtain good correspondence with experimental data over a wide range of coupling strengths. Previous studies have also reported realistic functional connectivity without including plasticity or introducing other heterogeneities into the model [1,12]; and consistent with these studies, we find here that ISP is not essential for our model to achieve good correlation for an *individual* measure of functional connectivity. However, we are only able to simultaneously achieve high correlation with *multiple* measures of functional connectivity (including AEC, PLV and PLI) with the inclusion of ISP. Our results complement previous work indicating that locally balancing excitation and inhibition results in more realistic dynamics [13,23]. Notably, we have obtained similar results with online ISP to previous work using offline FIC, using a different neural model, a different imaging modality, and with the inclusion of delays. The similarities in outcome across these studies suggest that our results reflect a more general process that could translate to other models as well.
3. *ISP enables synchrony/metastability to be related to previous Kuramoto model findings*. Previous studies used simple phase oscillators to show how fundamental synchronization dynamics can at least partially explain functional connectivity [1]. We can reproduce the same type of dynamics in our more realistic model, but only when excitation and inhibition are balanced. Without ISP, we obtain similar results to previous work using the Wilson-Cowan model with unbalanced excitation but without delays, where the oscillatory properties of the network strongly depend on coupling strength [15]. When balanced is restored by ISP, this sensitivity is greatly reduced, and we find a high synchrony regime with strong coupling and short delays, a low synchrony regime with weak coupling, and an intermediate metastable regime that gives rise to realistic functional connectivity, consistent with previous work [1,73].
4. *The dynamics are robust to moderate changes in the ISP target firing rate*. When we lowered the ISP target enough to produce a qualitative change in dynamics, the model lost the ability to produce realistic functional connectivity, demonstrating that simply balancing excitation and inhibition is not sufficient to produce realistic dynamics. The correct choice of activity level is also important, although we found that the dynamics are not overly sensitive to the choice of target activity level in the oscillatory regime. When we doubled the target level of activity, we obtained qualitatively similar results, but shifted to shorter delays for the same coupling strength.
5. *Coarsely balancing excitation and inhibition yields qualitatively similar behaviour to ISP*. We find that coarsely balancing excitation and inhibition with homogeneous inhibition in the network can produce qualitatively similar results to ISP. Performance in functional connectivity measured by PLV and PLI was comparable to the full ISP simulations, but performance in AEC was somewhat reduced. These results suggest that the reduced sensitivity to coupling strength and the qualitative similarity between Fig 4B and Fig 9A can be primarily explained by the overall global balance of excitation and inhibition. Unlike the local balance produced by ISP, the coarse balancing examined here is an offline optimization without any associated physiological mechanism. However, there are a range of plasticity mechanisms that operate on a variety of spatial and temporal scales [74] that could potentially serve as the physiological mechanism for such a process. Previous work has investigated the use of signals encoding large-scale network activity to control small-scale plasticity [75] which could bridge global and local scales. Similarly, there is evidence that ISP can interact with excitatory plasticity [76], which also opens the possibility of controlling homeostasis at larger spatial scales owing to the long range of excitatory projections.

### Comparing model and data functional connectivity

There is considerable freedom in how to quantify similarity between model and data functional connectivity. A natural choice is to correlate the unique parts of the connectivity matrices, but even this is a nontrivial decision when working with MEG/EEG because the connectivity profiles are frequency band specific. Because the relatively simple model we used here does not predict band-specific connectivity, in this study we avoid the issue of combining connectivity across frequency bands by focusing only on the alpha band. We used the amount of individual variability in functional connectivity to provide an interpretation of the similarity in functional connectivity between model and data. The anatomical connectivity matrix we used in this study was an average across multiple subjects computed from the HCP dataset, and comes from different subjects to those used for our estimates of functional connectivity. The functional connectivity from the model is analogous to an individual estimate of functional connectivity, and for an ideal biophysical model we would expect that the simulated functional connectivity should not be significantly less correlated with the group average connectivity than any real individual. We found that in all connectivity measures, the model performance was not significantly different to individual variability. Interestingly, the model’s similarity for AEC is lower than the mean individual similarity regardless of model parameters, whereas in the optimal parameter regime, the model outperforms individual similarity in data for PLV and PLI. This likely reflects the relative dominance of different sources of variability in the data—AEC is known to be a more reproducible measure than PLV and PLI both within and across subjects [63,77], which suggests that there is relatively more noise in PLV and PLI than AEC. This results in a reduction in similarity between single-scan estimates of individual functional connectivity and the group average for those metrics.

Having achieved model performance consistent with individual variability where the metric is the correlation between functional connectivity matrices, future work may focus on increasing similarity in other metrics, rather than further improving correlation. For example, the Kolmogorov-Smirnov distance between the distributions of values in the connectivity matrices would quantify the difference in magnitude of connectivity, which is at present quite large (as shown in Fig 5, particularly for PLV and PLI).

### Effect of orthogonalisation

In this study, we applied the same analysis pipeline to the model predictions and to the data, including spatial leakage correction by signal orthogonalisation. Including an orthogonalisation step to compensate for source leakage is an integral part of most MEG analysis pipelines, to eliminate beamformer-induced connectivity [62,63,67,77–79]. This procedure may also remove some genuine neuronal connectivity, but how much genuine neuronal connectivity is removed cannot be determined based on the experimental data alone. In the model, by design all functional connectivity can only be neuronal in origin. However, it is possible to produce functional connectivity in the model that would not survive orthogonalisation–for example, the highly-synchronized regime has strong zero lag correlations that are greatly affected by orthogonalisation. By including orthogonalisation in our analysis, we ensure that the model predictions arise through mechanisms that are compatible with the experimental data. Future work could also investigate using a forward model to simulate MEG sensor timecourses, whether to compare activity to data in sensor space where spatial leakage correction is not necessary, or to then use a beamformer step to introduce realistic spatial leakage into the model signals.

### Zero-delay functional connectivity predictions

We also investigated the effect of changing the target activity level, and found that with the higher ISP target, the model could exhibit high similarity to experimental data even with zero delay. This is an interesting finding because some studies do not include propagation delays [8,14,80] and still find realistic functional connectivity, while others find that delays are required to obtain realistic model predictions [1,12,81]. Our model is able to exhibit both kinds of behaviour, depending on the target level of activity. In this study, we have leveraged the fast timescale of MEG to also examine phase-based metrics of functional connectivity. There are significant propagation delays in brain networks, and although the delays may not be critical when modelling fMRI, they are important in MEG and EEG because they are on a similar timescale to oscillations in neural activity. Phase metrics are expected to be sensitive to propagation delays between brain regions, and intuitively this suggests that the optimal parameter regime in the model should have a nonzero delay. However, for the high ISP target simulation at zero delay, the model is still fairly well correlated with data even in PLV and PLI.

### Model selection and intrinsic oscillatory frequency

There are contrasting approaches in previous studies regarding the selection of intrinsic oscillatory frequency for uncoupled brain regions. A key factor is whether frequency suppression occurs in the model when brain regions are coupled [1,82]. For models that display frequency suppression such as the Kuramoto model, individual brain regions typically have an intrinsic frequency in the gamma band [1], while models that do not use oscillators with intrinsic frequencies in bands of interest e.g. alpha [8] or otherwise examine functional connectivity by incorporating a hemodynamic response function that reduces dependence on oscillatory frequency [12]. The former approach is motivated by both the hypothesis that isolated neural masses resonate in the gamma band, and by the fact that frequency suppression would otherwise shift oscillations to even lower frequencies (e.g. intrinsic alpha oscillations becoming network delta oscillations). The latter approach advocates that due to other connectivity such as thalamocortical interactions, sufficiently large brain regions may oscillate intrinsically at much lower frequencies. In this study, we used a model with local oscillations in the alpha band, because for the Wilson-Cowan model we do not see large frequency suppression at the network level for parameters that produce realistic functional connectivity. This alpha band timescale is much faster than fMRI, although there are even faster dynamics that can be investigated with MEG. A related open question is how to produce different band-specific patterns of functional connectivity using a single anatomical connectivity matrix. One promising direction may be to introduce multiple timescales of dynamics into the local model–for example, by introducing additional populations with different intrinsic oscillatory frequencies [41], having the local effective time constants depend on network properties [14], or by using a conduction-based neural mass model [83,84] that incorporates multiple timescales through the inclusion of multiple receptor types, each with a different time constant.

### Alternate routes to balanced networks

We have examined inhibitory synaptic plasticity due to its biological plausibility as a mechanism for balancing excitation and inhibition, but it is not the only possibility. Because the fundamental imbalance in excitation and inhibition in this study originates with the fact that we only include long-range E-E connections, an obvious question is whether E/I balance could be achieved simply by including long-range E-I connections as well. The clear proportionality between long-range excitation and local inhibition suggests that this may be possible to some extent, and including E-I connections based on the same anatomical connectivity matrix will naturally mean that the increase in input to the inhibitory populations is proportionate to long-range excitation. However, balancing excitation and inhibition would likely require different global coupling strengths *C* for the E-E and E-I connections, and this in turn would necessitate some sort of optimization or homeostatic mechanism to tune them. Thus it is unlikely that including long range E-I connections would eliminate the need for a homeostatic mechanism. Similarly, balancing excitation and inhibition through long range E-I connections requires that these connections be proportionate to long-range excitation, but achieving this balance in connection strengths in the real brain would likely require its own regulatory mechanism. Finally, there are also other mechanisms that could regulate excitatory activity–for example, intrinsic plasticity that directly modulates the excitability of the excitatory populations [85,86]. These may offer alternate routes to balancing excitation and inhibition at the network level.

### Future work

One of the proposed roles of ISP is to oppose perturbations to the network, improving the robustness of dynamics to changes such as lesions. Previous studies have modelled lesions by disconnecting regions from the network and examined the effect this has on network [3,6,73,87]. In some cases, removing a region can dramatically change oscillatory activity in the network. Does balancing excitation and inhibition restore functional connectivity? There is evidence that offline balancing of inhibition is able to restore functional connectivity as measured by fMRI [88]. It is an open question whether the same is true for more biophysically plausible online homeostatic mechanisms, although the results presented here suggest that the outcome may be comparable.

Aside from frequency-specific functional connectivity, there is also emerging evidence that changes in oscillatory frequency are linked to connectivity and functional hierarchy, with areas higher in the hierarchy exhibiting lower frequency oscillations [89–91]. In a previous study, a similar model to the one used here was able to produce this behavior through an upscaling of excitatory inputs depending on the region’s position in the hierarchy [14]. This upscaling affects different local connections to ISP, and could therefore be implemented in conjunction with ISP. Intuitively, ISP should act to oppose the upscaling of excitation, but whether this will eliminate the frequency suppression effect is unclear. More generally, this points to a broader question of the interaction and potential interference between homeostatic mechanisms and their effect on desirable network inhomogeneities.

In the present study, we approximated the delays in the network using Euclidean distance and a uniform conduction velocity, which matches previous recent work [1,65,73,92]. The effect of delays in brain networks and the sensitivity of dynamics to a distribution of delays is still an open question [93–97]. Specifically, the effect of tract length and myelination, both of which affect the propagation delay between brain regions, is yet to be explored. The framework developed in this study is suitable for investigating alternate methods for calculating delays, and we plan to investigate the effect of accounting for tract length and myelination in future work.

In this study, we focused on reproducing static functional connectivity in the model. Having reached levels of performance within the range of individual variability in data, future work on resting state connectivity may focus on reproducing more features in the data rather than pursuing higher correlations. In particular, the nonstationary nature of real brain dynamics is well established [78,98,99], and there have been a range of recent developments in methods to quantify fast transient dynamics with regard to functional connectivity in electrophysiological data [21,100–102]. What network properties are responsible for these dynamics is still an open question, although there a wide range of possible mechanisms that produce transient dynamics in models [1,9,55,103–105]. The next step in this direction is to examine transient states in our model in more detail, to characterize properties such as the statistics of state transitions, and the transient patterns of functional connectivity and frequency content.

## Supporting information

S1 Supplementary Material Additional supplementary results referenced in text. (PDF)

S1 Text Parcel labels. Maps ROI index to brain region.
(TXT)

S1 Data Parcellation NIFTI file. (GZ)

S2 Data Connectivity matrix. Values for matrix shown in **Fig 1C**.
(TXT)

S3 Data Distance matrix. Values for matrix shown in **Fig 1D**.
(TXT)
