# Supplementary material for: A biophysical model of dynamic balancing of excitation and inhibition in fast oscillatory large-scale networks
Source: PLoS Comput Biol. 2018 Feb 23;14(2):e1006007. doi: 10.1371/journal.pcbi.1006007 (PMC5841816; doi:10.1371/journal.pcbi.1006007)
Supplement: S1 Supplementary Material — (PDF) [file pcbi.1006007.s001.pdf]

A biophysical model of dynamic balancing of excitation and inhibition in fast oscillatory large-scale networks

S1 Supplementary Material

Band specific connectivity

As mentioned in the main text, the functional connectivity pattern in each harmonic peak is very similar to the functional connectivity in the fundamental peak, hence we focus on connectivity only in the alpha band in the present study. **Figure A1** shows the functional connectivity profile in three different bands, each approximately centred on one of the peaks, for the same model parameters used in **Figs. 5** and **7**. For the bands marked in **Fig. A1a**, the corresponding AEC profiles are shown in **Figs. A1b-A1d**.

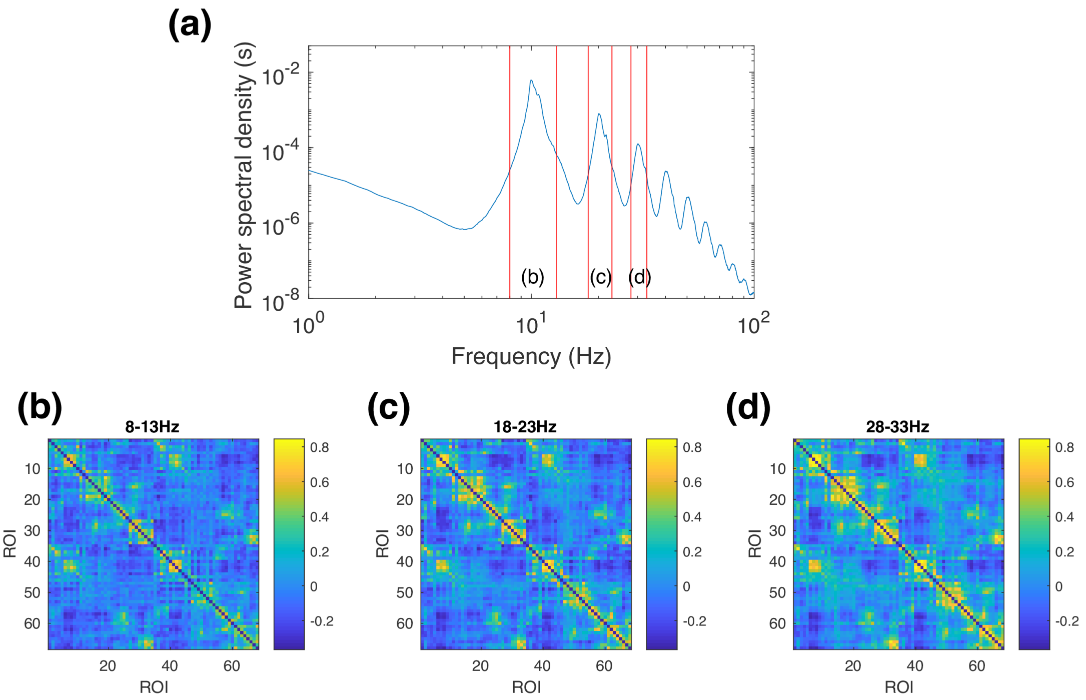

Fig. A1 Functional connectivity profiles in different frequency bands. Connectivity profiles are shown for model parameters used in Figs. 5 and 7. (a) Frequency spectrum of excitatory activity averaged over brain regions (blue). The red lines mark the frequency bands used for analysis. (b-d) Functional connectivity profiles computed using AEC for each of the bands marked in panel (a).

Defining ISP convergence

By definition, plasticity ceases when $\frac{dc_{ie}(t)}{dt}$ is zero. Hence, the convergence or success of ISP would normally be quantified by comparing the target activity rate to the instantaneous excitatory activity:

$$\rho = E(t)$$

However, in this study we will be operating the model within an oscillatory regime. As a result, $\frac{dc_{ie}(t)}{dt}$ will vary over the period of an oscillation, such that on electrophysiological timescales, the system oscillates between being above and below the target activity level. Instead of assuming the system has converged when $\frac{dc_{ie}(t)}{dt}$ is zero, we consider the system to have converged if dc_{ie} is zero integrated over a period spanning several oscillations, which implies there is no sustained change in c_{ie} over time. Integrating both sides of Eq. (4) yields

$$\tau_{isp} dc_{ie} = \int I(t)E(t)\,dt - \rho \int I(t)\,dt\,, \tag{A1}$$

and setting $dc_{ie} = 0$ provides the requirement for convergence as

$$\rho = \frac{\int I(t)E(t)\,dt}{\int I(t)\,dt}\,. \tag{A2}$$

Note that Eq. (A2) corresponds to a weighted average of excitatory activity. A further subtlety to consider is what happens when the system is operating at a point where it produces sustained nonlinear oscillations. Since inhibitory activity in the model is driven by local excitatory activity, $I(t)$ is largest when $E(t)$ is also large. The weighted average in (A2) therefore weights times with high excitatory activity more strongly, and an *unweighted* average of excitatory activity over this same time would be smaller than the expression in (A2). In these cases, the system will thus converge to a state where that the average excitatory activity is smaller than ρ.

Robustness to noise

To demonstrate the robustness of dynamics and functional connectivity to noise, **Figure A2** shows simulations using the same delay/coupling parameters as used in **Fig. 5**, but with different levels of noise. Note that we included the ISP stage of the simulation as well, to verify that ISP is also robust to the choice of noise signal. For these simulations, the noise signals were sampled from Gaussian distributions with zero mean and standard deviations of 0, 0.05, 0.1, and 0.2, which correspond to no noise, and to 5, 10, and 20 times the original amount of noise. The results are qualitatively very similar even with a 10-fold increase in noise, although larger differences begin to emerge after this point. The phase-based connectivity metrics are more sensitive to noise than AEC, but **Fig. A2** shows that the magnitude of the phase connectivity metrics is still highly robust to a moderate increase in noise. This provides confirmation that our results primarily reflect the nonlinear dynamics of the model, rather than the noise process.

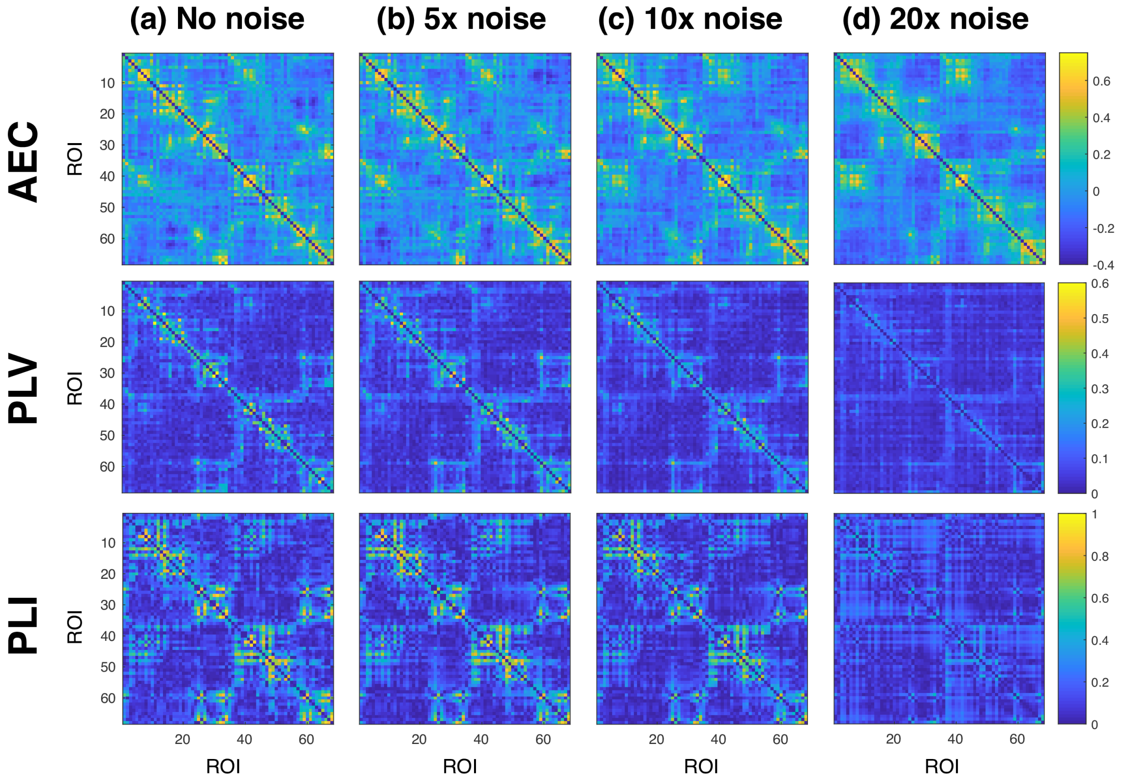

Fig. A2 Functional connectivity metrics for different noise levels. Functional connectivity in each metric is shown in the model for the parameters marked by the red dot in **Figs. 4, 6,** and **7**, with varying levels of noise. The noise levels are (a) no noise (b) standard deviation 0.05 (c) standard deviation 0.1, and (d) standard deviation 0.2.

Synchrony with orthogonalisation

As synchrony and metastability are affected by spatial leakage, it is necessary to include an orthogonalisation step when comparing them to the model. The synchrony and metastability matrices shown in **Figure A3** are from the same simulations shown in **Fig. 6c** and **6d**, except that the parcel activity timecourses were orthogonalised prior to estimating the phase timecourses. Experimental estimates of synchrony and metastability should be compared to **Fig. A3** rather than **Fig 6.** Orthogonalisation reduces synchrony and metastability in most parts of the parameter space we examined. As expected, the effect is most pronounced in the high synchrony regime, because strong phase synchronization results in large zero-lag correlations that the orthogonalisation process is designed to remove. Thus, the clearly visible high synchrony region in upper right part of **Fig. 6c** is entirely absent from **Fig. A3a**. For the parameters in the optimal regime marked by the red dot, the synchrony was 0.5292 without orthogonalisation and 0.2075 with orthogonalisation. The metastability was 0.2306 without orthogonalisation and 0.1052 with orthogonalisation. In comparison, for our experimental data set, the group-average alpha band synchrony was 0.1079 and the metastability was 0.0563. Note that there are sources of noise present in the experimental data that are not present in the model, which would likely result in a reduction in synchrony.

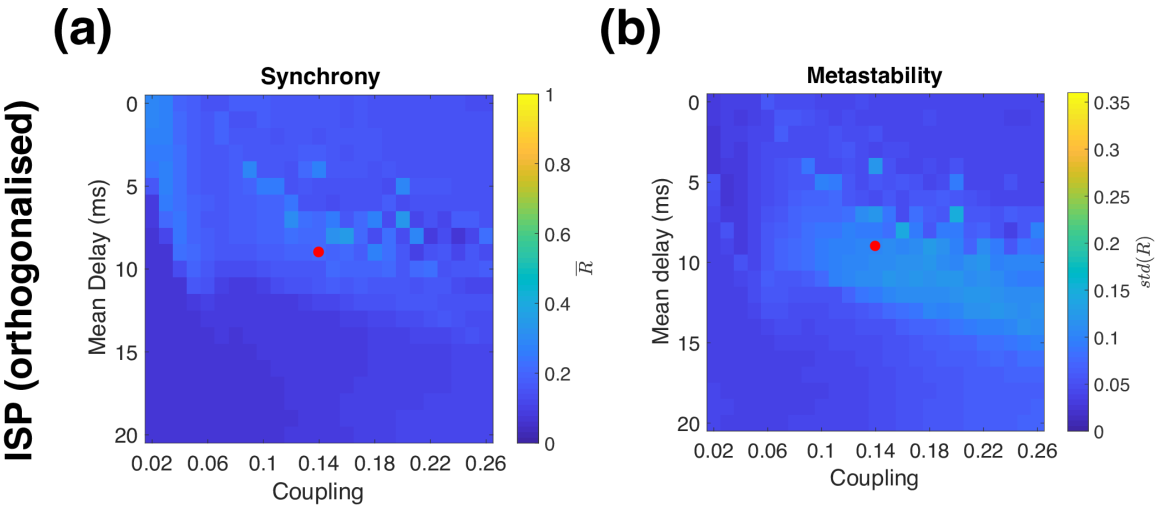

Fig. A3 Synchrony and metastability with orthogonalisation. (a) Alpha band synchrony (averaged over time) (b) metastability (standard deviation of synchrony), in the model with ISP and with orthogonalisation. The red dot corresponds to the parameters shown in **Figs. 5** and **7.**

Synchrony for high and low ISP targets

Synchrony and metastability for the simulations in **Fig. 8** are shown in **Fig. A4**.

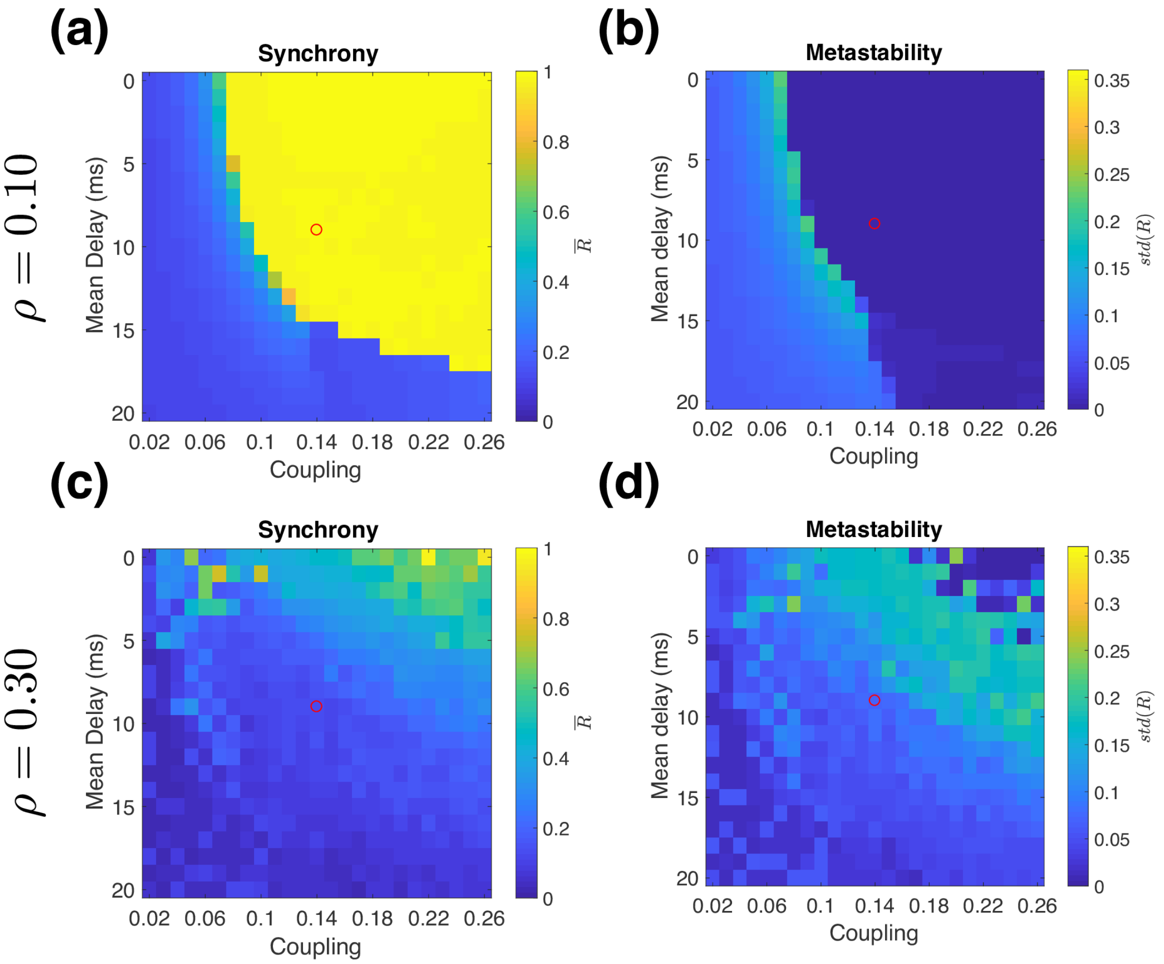

Fig. A4 Synchrony and metastability. Alpha band synchrony (averaged over time) and metastability (standard deviation of synchrony), in the model for (a,b) low and (c,d) high ISP targets. The open circles correspond to the representative optimal delay and coupling values shown in **Fig. 4** and used in **Figs. 5** and **7.**

ISP convergence in brain simulations

To investigate the convergence of ISP, we examine the standard deviation of c_{ie}, after providing enough time for the system to converge if possible. **Figure A5a** shows the standard deviation of c_{ie} computed over the final 500 seconds of the simulation (discarding the first 1000 seconds), averaged over brain regions. We consider three regimes – a highly synchronous regime with low delay and strong coupling, a metastable regime that gives rise to realistic functional connectivity, and a low synchrony regime with weak coupling and long delays. The mean activity and plasticity timecourses for example parameters in each regime are shown in **Figs. A5c-A5h**.

We first examine the discrepancy in activity that gives rise to ongoing plasticity. **Fig. A5b** shows the mean I-weighted E activity **(Eq. A1)** in each brain region, sorted by value. For the low synchrony and metastable parameter regimes, all brain regions have activity close to the target value. However, for the high synchrony parameters, although a small number of brain regions can reach the target level of activity, some exhibit higher levels of activity, while others exhibit lower levels of activity. The corresponding activity time series are shown in **Figs. A5c-A5e**. The high synchrony regime has sustained differences in mean activity across the network, whereas the metastable and low synchrony regimes have variable levels of activity that are balanced across the network. Importantly, the asymmetry in activity in the high synchrony regime is robust to moderate changes in c_{ie}. **Figs. A5f-A5h** show the corresponding plasticity time courses for the three parameter regimes. In the high synchrony regime, there are large ongoing changes in synaptic strength. For example, inhibition in some brain regions decreases over a long period of time (e.g. the bottom-most trace, which has an ongoing decrease in inhibition from 500s onwards), which indicates that although inhibition in that brain region is decreasing, the amount of activity is not increasing.

Our interpretation of these results is that the high synchrony regime exhibits dynamics that are strongly dependent on the long-range connectivity and the network, resulting in the asymmetries in activity being robust to moderate changes in synaptic strength in parts of the network. In contrast, for the weakly coupled network it is possible to optimize the mean level of activity in each brain region independently, due to the weaker effect of the rest of the network. This enables each region to independently achieve a local balance between excitation and inhibition. The metastable regime shown in **Fig. A5g** lies somewhere in between. On short timescales (on the order of 0.5-2 seconds), there are asymmetries in activity, but these are not stable and continuously change. The network can be considered to transiently visit different asymmetric oscillatory states. However, on long timescales that average over these states, the metastable regime can achieve the target level of activity in all brain regions, as shown in **Fig. A5b**. We note that because convergence in the metastable case requires averaging over transient states, it is especially important to ensure that the plasticity timescale τ_{isp} is slow enough that the synaptic strengths are approximately constant for the duration of each transient state, which is considerably longer than the oscillatory period of the units (~0.1 seconds).

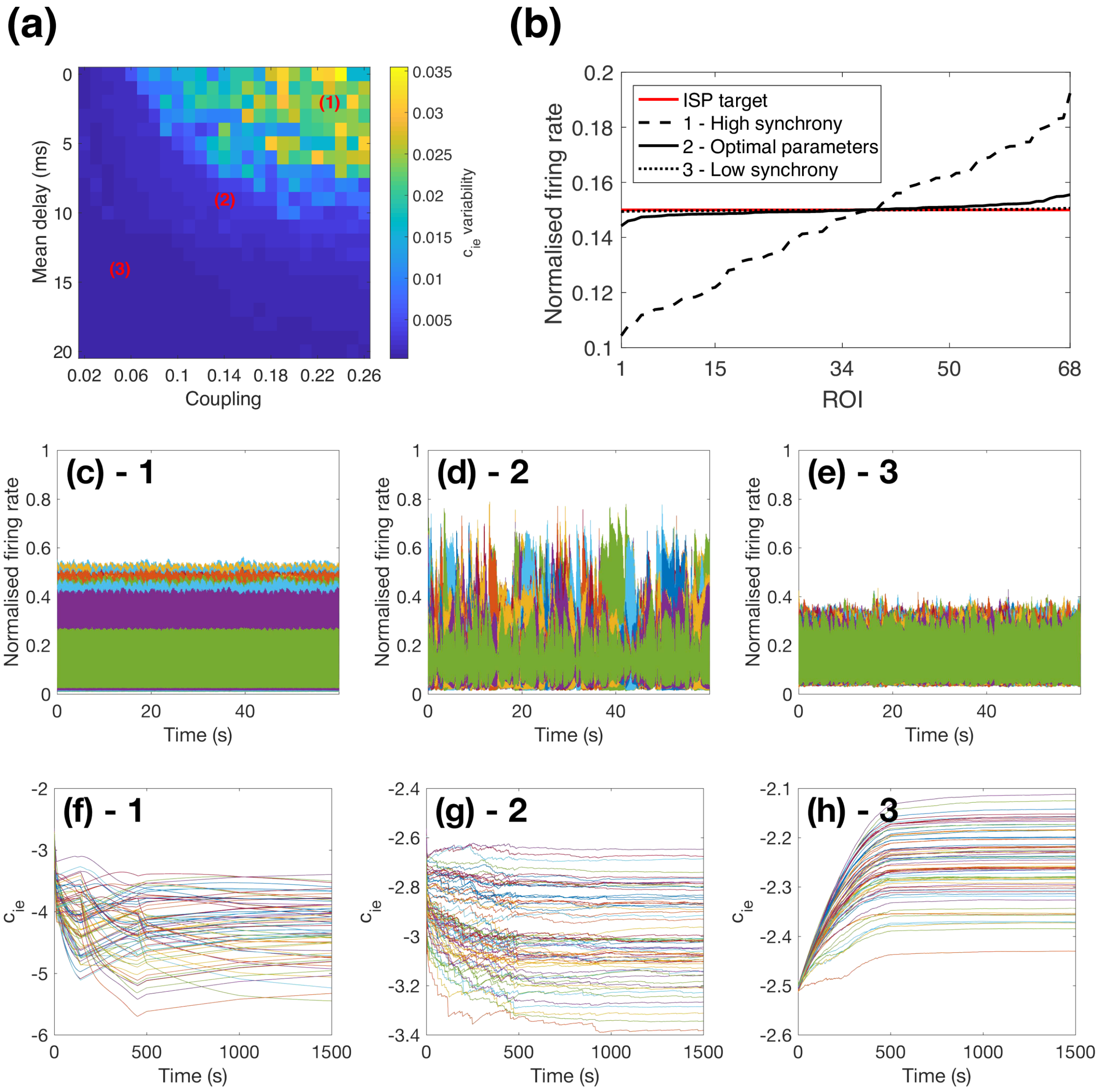

Fig. A5 ISP convergence for different delay-coupling parameter values. (a) Variability in $\boldsymbol{c_{ie}}$ for the final 500 seconds of the plasticity simulation runs. (b) Difference between ISP target and actual I-weighted excitatory activity in each brain region, for the three parameter combinations shown in Fig 9. Brain regions have been sorted by difference in activity. (c-e) Excitatory activity in each brain region for the three parameter regimes marked in (a). (f-h) Time series showing changes in $\boldsymbol{c_{ie}}$ due to ISP for the same 3 regimes.

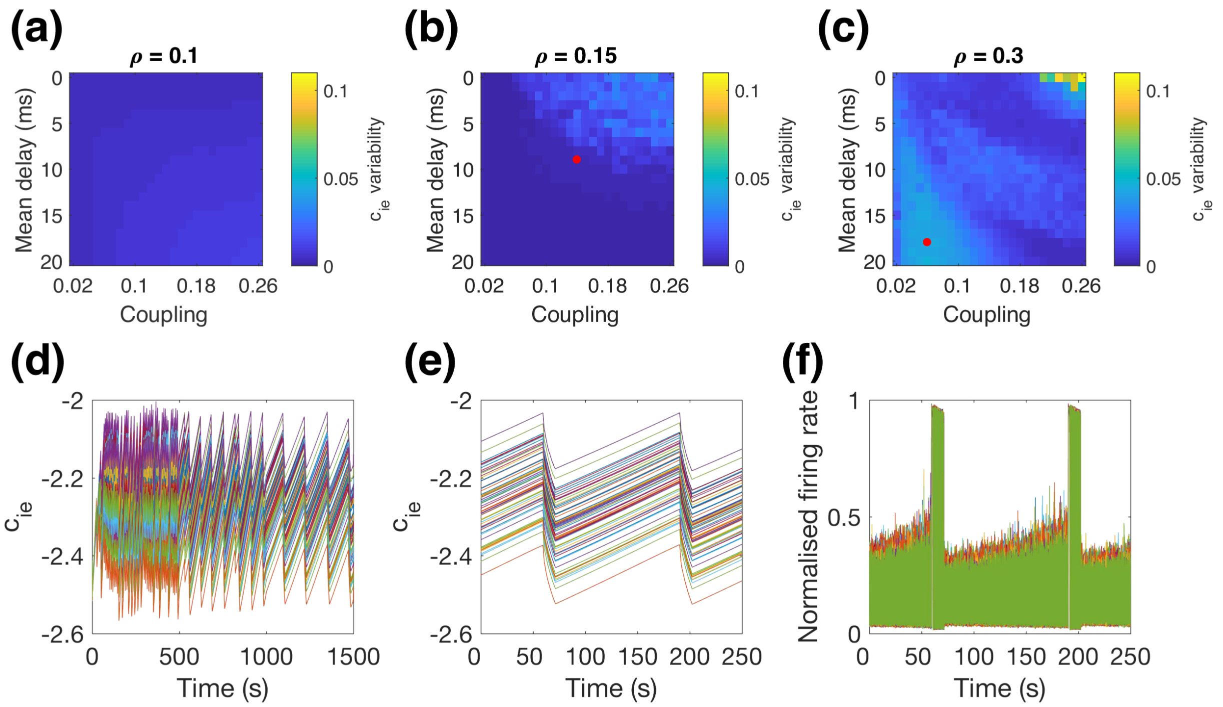

Fig. A6 ISP convergence for different ISP target activity levels. Variability in $\boldsymbol{c_{ie}}$ for the final 500 seconds of the plasticity simulation runs, for (a) Low-activity ISP target $\boldsymbol{\rho = 0.1}$ (b) Moderate-activity ISP target $\boldsymbol{\rho = 0.15}$. The red dot indicates the optimal parameters shown in **Figs. 5** and **7** (c) High-activity ISP target $\boldsymbol{\rho = 0.3}$. The red dot indicates the parameters shown in panels (d)-(f). (d) Timecourse of $\boldsymbol{c_{ie}}$ in initial simulation with variable $\boldsymbol{\tau_{isp}}$ (e) Short simulation showing periodic change in synaptic strength. (f) Corresponding excitatory activity at the same times as panel (e).

Next, we investigated the convergence properties of ISP when changing the target activity level ρ. **Figs. A6a-A6c** show the variability in c_{ie} over the final 500 seconds of the simulations, for the low, medium, and high activity targets. For the low target activity $\rho = 0.1$, ISP can achieve the target firing rate regardless of the long-range delay and coupling. For the high activity, ISP target $\rho = 0.3$, variability in c_{ie} is small for the parameter regime that best matches experimental data. However, ISP no longer converges in the weakly interacting regime, with long delays and low couplings. **Fig. A6d** shows the timecourse of c_{ie} for the parameters marked by the red dot in **Fig. A6c**. There are large ongoing oscillations in synaptic strength. The amplitude of these oscillations does not change when the plasticity rate is decreased (at 500s and 1000s), which indicates that it is not the result of coupling between neural activity and plasticity. **Figs. A6e** and **A6f** show a detailed segment of the fluctuation in c_{ie} and the corresponding excitatory activity. For these parameters, high inhibition is

associated with low activity, low inhibition with high activity, and there is a bistable regime where both the low activity and high activity dynamics are possible with the same level of inhibition. Initially, the mean activity is below the ISP target in all populations, which causes inhibition to decrease. However, a bifurcation is encountered at which point the amplitude of the oscillations in the network suddenly increases, and the mean activity becomes much higher than the ISP target. In response, inhibition begins to increase again. However, the high amplitude oscillation does not lose stability immediately and when the network transitions back to a low activity state, the mean activity is well below the ISP target. Plasticity thus moves the system periodically over the bistable regime, reversing direction whenever one of the activity patterns becomes unstable, because the ISP target lies between the high and low activity regimes.

In summary, we have identified two distinct cases where ISP fails to converge – a case where strong global interactions prevent all of the units from achieving the same level of mean activity, and a case where a periodic oscillation in synaptic strength occurs if the ISP target lies between bistable high and low activity states. For the parameters that we tested in this study, convergence was not a problem in the parameter regimes that give rise to realistic functional connectivity, but there are clearly restrictions on the level of synchronization and on the ISP target if the synaptic strengths are to converge.

It is a possibility that the periodic changes in synaptic strength are only a local minimum solution, and that a different initialization for the synaptic strengths could result in convergence of ISP. Due to the computationally intensive nature of the ISP simulations, a practical approach for testing this would be to use an offline optimization mechanism to first check for the existence of a stable solution for plasticity with the desired activity level, as this is a necessary requirement for ISP convergence. Even this approach is challenging – the parameter space is high-dimensional with as many synaptic strengths to optimize as brain regions, and we have observed that in the optimal parameter regime, the synaptic strengths are strongly correlated but not precisely correlated with long-range excitation (**Fig 7b**), which means that the problem cannot be reduced to a two-parameter optimization of the line shown in in **Fig 7b**. The cost function would also be extremely expensive compared to those used for similar previous work (Deco et al., 2014) because the transient states in the optimal parameter regime require that simulations be run for a sufficiently long time to enable averaging over the states, and delays need to be included. Assessing the stability of a

plasticity solution, if one is found, is also challenging because whether plasticity causes perturbations in synaptic strength to grow or decay depends on the highly nonlinear fast dynamics in the model. Regardless, offline optimization algorithms will also need to account for the possibility that there may be no stable solutions. In contrast, the online learning rule we have investigated in this study is simpler to investigate and more biologically relevant, with the trade-off that we do not exhaustively explore the space of synaptic strengths.
